# A comparative analysis of the epidemiology of influenza and respiratory syncytial virus in Russia, 2013/14 to 2018/19

**DOI:** 10.7189/jogh.12.04009

**Published:** 2022-02-05

**Authors:** Saverio Caini, Kirill Stolyarov, Anna Sominina, Elizaveta Smorodintseva, Lisa Staadegaard, John Paget, Daria Danilenko

**Affiliations:** 1 Netherlands Institute for Health Services Research (Nivel), Utrecht, the Netherlands; 2 Smorodintsev Research Institute of Influenza, Saint Petersburg, Russia

## Abstract

**Background:**

Influenza and respiratory syncytial virus (RSV) are among the leading causes of lower respiratory tract infections worldwide. We conducted a comparative analysis of the age distribution and spatiotemporal epidemiology of influenza and RSV in Russia using sentinel surveillance data from 2013-14 to 2018-19 in six cities located in the western, central, and eastern regions of the country.

**Methods:**

We calculated the positivity rate for influenza and RSV (by month, season, and overall) in each city, separately for patients seen at the primary and secondary care level (out-patients medical centres housing GP practices and infectious diseases hospitals, respectively). We compared the age distribution of patients infected with the different influenza virus (sub)types and RSV.

**Results:**

A total of 17 551 respiratory specimens were included: the overall positivity rate was 13.5% for influenza and 4.4% for RSV. The A(H1N1)pdm09, A(H3N2) and B virus (sub)types caused 31.3%, 44.0% and, respectively, 24.7% of all influenza cases. The median age was older among influenza (15 years) than among RSV patients (3 years); differences across influenza virus (sub)types were seen only at the primary care level, with influenza A(H3N2) patients being significantly older than A(H1N1)pdm09 or B influenza patients. The timing of influenza epidemics was similar across cities, with the peak typically occurring in February or March. In contrast, the typical peak timing of RSV epidemics varied largely across cities, and the virus was often detected also in spring and summer months (unlike influenza).

**Conclusions:**

Influenza and RSV epidemiology differed in many regards in Russia, especially in the timing of epidemics and the age distribution of infected subjects. Health policies aimed at containing the burden of diseases of viral respiratory infections in Russia should take these findings into account.

Influenza viruses and respiratory syncytial virus (RSV) are among the leading causes of Lower respiratory infections-associated burden of disease [1,2]. In general, a thorough knowledge of influenza and RSV epidemiology across age groups and health care settings, and in particular of the spatial and temporal dynamics of their seasonal epidemics, is a pre-requisite for countries to enhance the effectiveness of public health interventions aimed at reducing their disease burden (eg, influenza vaccine, palivizumab (PVZ) prophylaxis, and hospital preparedness and response). These considerations are especially valid in Russia, a vast country extending over several climate zones and with considerable internal variability in health-relevant conditions (eg, socio-economic status, access to health care, and risk factors for infection and severity) coupled with a rapidly aging population [3-5]. This poses tremendous challenges to deploy timely and effective preventive measures and means there is need for coordinated and sustained efforts to prevent influenza and RSV in Russia.

Influenza vaccination campaigns have been introduced in Russia since the mid-1990s. Influenza vaccination is offered free of charge for everyone and prioritized for infants, children, pregnant women, and the elderly, as well as health care workers and those occupied in education, transport and trading [6]. Vaccination coverage has increased from 46.6% in 2017 to 59.9% in 2020 [7-9]. The vaccination campaign starts in late August and ends in December. The early start of the immunization campaign is due to the large number of vaccine doses that need to be deployed across the vast territory, and is aimed at vaccinating schoolchildren and teachers before the school year starts (September 1st). All influenza vaccines are inactivated with more than 99% of them being produced in Russia. Quadrivalent inactivated vaccines were approved in 2019 and are gradually replacing the trivalent vaccines according to the national plan of the Federal Service for Surveillance on Consumer Rights Protection and Human Well-being (“Rospotrebnadzor”) and the Ministry of Health [10,11].

Russia has established a syndromic surveillance system for viral respiratory infections based on multiplex PCR assays. Here, we used surveillance data to conduct a comparative analysis of the epidemiology of influenza and RSV during six consecutive seasons (from 2013-14 to 2018-19) in six cities around the country. The focus of the present analysis aims to assess which age groups were most frequently affected by either virus, and to determine whether the timing of seasonal epidemics differed between viruses and geographically across the country, with both aspects being important for prevention and control measures.

## METHODS

### Surveillance of respiratory virus infections in Russia

Routine influenza surveillance was initially established in Russia in the late 1960s. Russia actively participates in the WHO Global Influenza Surveillance and Response System (GISRS) with two National Influenza Centres (NIC) located in Moscow (since 1952) and Saint Petersburg (since 1971), and with the WHO H5 Reference Laboratory in Novosibirsk [12]. The surveillance system collects weekly data on incidence, hospitalisations and deaths along with laboratory confirmation for influenza, RSV, and other respiratory viruses. Following the 2009 pandemic, the sentinel surveillance in Russia was divided for outpatients (influenza-like illness (ILI) cases) and hospitalised patients (severe acute respiratory infections (SARI) cases) according to the WHO recommendations and approved by the Rospotrebnadzor, and upgraded in 2010 to meet the recommendations of the WHO Regional Office for Europe.

The influenza sentinel surveillance system is operated in ten cities across the country. Each city monitors ILI and SARI cases year-round according to the WHO/Europe case definitions [13]. Individual electronic case reporting forms (eCRF) are completed by clinicians under the supervision of the chief epidemiologists at each site, and then integrated into a unified national database, which include information on patients’ demographics, influenza vaccination status, comorbidities, clinical symptoms, course of disease, and disease outcome.

In the present study, we distinguished patients into those swabbed at the “primary” (ILI) and “secondary” (SARI) care level (Table S1 in the **Online Supplementary Document**). The primary care level mostly corresponds to polyclinics, ie, multidisciplinary outpatients medical centers that house general practices as well as several specialists’ services and diagnostic facilities. At the primary care level, only a subset of ILI patients (2 or 3 per week, depending on the site) presenting on the first three days of a week are swabbed. Most influenza and RSV cases seen at the secondary care level come from specialized infectious diseases hospitals and (in lower percentages) other adult and paediatric hospitals. All patients presenting with SARI at the secondary care level are swabbed. Upon collection, nasopharyngeal swabs taken from both ILI and SARI patients are shipped to the NIC or its regional base laboratory and tested using rRT-PCR kits for influenza (Amplisens® Influenza virus A/B-FL, AmpliSens® Influenza virus A-type-FRT PCR kit) and for other respiratory viruses - RSV, parainfluenza, adenoviruses, seasonal coronaviruses, human metapneumoviruses, rhinoviruses, and bocavirus - (AmpliSens® ARVI-screen-FRT) [14,15]. The same diagnostic kits were used in all surveillance sites throughout the whole study period. Influenza A virus-positive samples were subtyped for A(H1N1)pdm09 and A(H3N2) viruses, while influenza B virus- and RSV-positives samples were not further subtyped due to the limitation of PCR assays registered in the country.

The six cities included in the present study (Kaliningrad and St. Petersburg in the Northwestern District, Lipetsk in the Central District, and Chita, Vladivostok, and Khabarovsk in the Far Eastern District) were selected out of the ten cities participating in the surveillance system (see Figure S1 in the Online **Supplementary Document**) after initial checking for the quality of the data in the database (regularity of the data and laboratory confirmation for all cases) and for the number of respiratory specimens tested during the study period (2013-14 to 2018-19). The four cities that were excluded (Moscow, Samara, Stavropol, and Novosibirsk) collected an average of fewer than 50 specimens per season (and had up to three seasons with no collected specimens) during the study period.

### Statistical analysis

We defined the positivity rate for influenza and RSV as the proportion of specimens testing positive for either virus. The positivity rate for influenza virus and RSV was calculated in each month, season (from July to June next year), and overall. We then calculated the proportion of influenza cases caused by the A(H1N1)pdm09, A(H3N2), and B virus (sub)type.

The age distribution was described using the median, inter-quartile range (IQR), and proportion of those aged ≥65 years. We then fitted simultaneous quantile regression models to compare the 10^th^, 50^th^ (median) and 90^th^ percentiles of the age distribution of subjects infected with influenza A(H3N2) (taken as reference), influenza A(H1N1)pdm09, influenza B, and RSV, separately for the primary and secondary care level. The choice to focus on three percentiles was motivated by the fact that the age distribution may present multiple modes and differ among patients infected with the different virus types and subtypes, and by the fact that being very young or very old is the most important determinant of severity for most respiratory infections. The analysis of the seasonality of influenza and RSV epidemics was conducted by first calculating (and depicting graphically using polar plots) the monthly average positivity rate. We then used the *monthglm* command (with a logit link) in R software (package “season”) to estimate the odds ratio (OR) and 95% confidence intervals (CI), of a respiratory specimen testing positive for influenza (or RSV) in each month compared to the peak month (taken as reference), defined as the month with the highest average positivity rate for that virus in that city [16]. The *monthglm* command fits a generalized linear model using a categorical month variable as independent variable, and is suitable when dealing with non-sinusoidal time series. An OR could not be calculated in those months (from late spring to early autumn) where no cases of influenza or RSV were reported.

### Ethics

Patients are included in the surveillance scheme after providing written informed consent to be swabbed and tested for respiratory viruses. In accordance with applicable laws, the retrospective analysis of anonymised data collected within respiratory virus surveillance needs no clearance of an Ethics Committee in Russia.

## RESULTS

Respiratory virus surveillance data were available from January 2014 to April 2019 (**Table 1**). The overall number of respiratory specimens was 17 551, distributed unevenly across cities (with St. Petersburg, Vladivostok and Chita contributing 72.1% of all specimens, while Vladivostok provided only 2.5% of them) and seasons (over 40% of specimens collected in 2016-17 and 2017-18). ILI patients from the primary care level contributed 66.7% of all specimens (from 32.6% in St. Petersburg to 100% in Khabarovsk). The overall proportion of ILI patients that were swabbed during the study period was 3.2% (from 1.3% in Kaliningrad to 7.1% in Lipetsk). The overall positivity rate for influenza and RSV was 13.5% and 4.4%, respectively: this varied both between surveillance sites and across seasons within each site (**Table 1**) and was generally higher when calculated among samples taken in the secondary vs primary care level (Table S2 in the **Online Supplementary Document**). The monthly number of respiratory specimens collected in each surveillance site, and of those testing positive for influenza or RSV, are shown in Figure S2 in the **Online Supplementary Document** (the positivity rate of other respiratory viruses is given in Table S3 in the **Online Supplementary Document****).**

**Table 1:** Seasonal number of respiratory specimens and their breakdown by level of care (primary and secondary, where influenza-like illness (ILI) and, respectively, severe acute respiratory infections (SARI) patients were tested), positivity rate for influenza virus and respiratory syncytial virus (RSV), and age distribution (median and inter-quartile range (IQR)) of subjects testing positive for influenza virus and RSV, in each surveillance site in Russia (seasons 2013/14 to 2018/19)

Federal District	Site	Season*	No. specimens	Level of care (%)				Influenza			RSV		
**Primary**	**Median age, years (range)**	**Secondary**	**Median age, years (range)**	**No. cases**	**Positivity rate (%)**	**Median age, years (range)**	**No. cases**	**Positivity rate (%)**	**Median age, years (range)**
**North Western**	**Kaliningrad**	2013/14	119	95.0%	17 (6-30)	5.0%	1 (0.3-3)	22	18.5%	11 (2-60)	9	7.6%	3 (<0.1-24)
		2014/15	173	98.3%	22 (4-32)	1.7%	2 (2-56)	8	4.6%	18 (8-56)	9	5.2%	6 (1-40)
		2015/16	217	94.0%	15 (4-34)	6.0%	0.6 (0.2-2)	26	12.0%	6 (0.2-51)	17	7.8%	5 (0.2-66)
		2016/17	319	81.2%	9 (4-24)	18.8%	4 (1-10)	88	27.6%	7 (0.6-55)	29	9.1%	3 (<0.1-45)
		2017/18	270	95.9%	7 (3-14)	4.1%	2 (0.4-3)	23	8.5%	5 (0.7-14)	23	8.5%	3 (0.2-33)
		2018/19	331	67.9%	6 (3-15)	32.1%	2 (0.8-4)	69	20.8%	5 (0.6-67)	56	16.9%	3 (<0.1-73)
		**Total**	**1429**	**86.1%**	**9 (4-26)**	**13.9%**	**2 (0.8-5)**	**236**	**16.5%**	**7 (0.2-67)**	**143**	**10.0%**	**3 (<0.1-73)**
	**St. Petersburg**	2013/14	314	31.5%	12 (9-15)	68.5%	23 (2-41)	54	17.2%	26 (0.8-89)	26	8.3%	1 (<0.1-66)
		2014/15	742	27.6%	13 (8-16)	72.4%	26 (2-43)	142	19.1%	32 (1-90)	26	3.5%	2 (<0.1-37)
		2015/16	612	36.4%	11 (6-15)	63.6%	27 (4-46)	123	20.1%	26 (0.7-78)	35	5.7%	6 (0.5-93)
		2016/17	708	32.2%	12 (9-16)	67.8%	22 (3-44)	97	13.7%	25 (1-89)	25	3.5%	2 (0.7-79)
		2017/18	808	31.9%	11 (7-15)	68.1%	30 (3-64)	134	16.6%	46 (<0.1-96)	32	4.0%	2 (0.2-71)
		2018/19	543	36.5%	10 (8-14)	63.5%	29 (4-59)	53	9.8%	25 (<0.1-92)	30	5.5%	3 (0.4-87)
		**Total**	**3727**	**32.5%**	**11 (8-15)**	**67.5%**	**26 (3-53)**	**603**	**16.2%**	**29 (<0.1-96)**	**174**	**4.7%**	**2 (<0.1-93)**
**Central**	**Lipetsk**	2013/14	378	75.1%	4 (1-11)	24.9%	1 (0.7-8)	88	23.3%	7 (<0.1-88)	1	0.3%	1 (-)
		2014/15	493	83.0%	7 (2-16)	17.0%	2 (1-15)	88	17.8%	14 (0.4-85)	9	1.8%	3 (0.7-17)
		2015/16	597	96.8%	3 (1-10)	3.2%	2 (0.7-15)	55	9.2%	21 (0.5-78)	16	2.7%	4 (0.3-69)
		2016/17	615	94.0%	3 (1-12)	6.0%	3 (1-42)	78	12.7%	10 (0.4-74)	25	4.1%	2 (0.2-53)
		2017/18	584	77.9%	3 (1-13)	22.1%	2 (1-7)	32	5.5%	13 (<0.1-80)	61	10.4%	1 (<0.1-61)
		2018/19	359	98.3%	3 (1-12)	1.7%	4 (2-55)	36	10.0%	6 (<0.1-67)	20	5.6%	2 (<0.1-36)
		**Total**	**3026**	**87.8%**	**3 (1-13)**	**12.2%**	**2 (1-12)**	**377**	**12.5%**	**20 (<0.1-92)**	**132**	**4.4%**	**2 (<0.1-69)**
**Far Eastern**	**Chita**	2013/14	431	52.4%	15 (4-33)	47.6%	38 (15-61)	75	17.4%	26 (1-80)	26	6.0%	3 (0.2-100)
		2014/15	714	63.0%	17 (6-30)	37.0%	23 (1-48)	72	10.1%	12 (0.5-84)	19	2.7%	6 (<0.1-88)
		2015/16	773	54.5%	17 (6-35)	45.5%	45 (23-62)	55	7.1%	23 (<0.1-90)	24	3.1%	4 (0.2-71)
		2016/17	811	53.1%	17 (6-37)	46.9%	42 (19-65)	86	10.6%	25 (0.5-92)	18	2.2%	2 (<0.1-69)
		2017/18	825	52.4%	18 (5-36)	47.6%	44 (19-63)	52	6.3%	17 (0.5-90)	38	4.6%	2 (<0.1-82)
		2018/19	609	59.0%	17 (6-33)	41.0%	28 (3-51)	60	9.9%	18 (0.7-71)	29	4.8%	4 (<0.1-76)
		**Total**	**4163**	**55.7%**	**17 (6-34)**	**44.3%**	**37 (15-61)**	**400**	**9.6%**	**20 (<0.1-92)**	**154**	**3.7%**	**3 (<0.1-100)**
	**Vladivostok**	2013/14	351	81.8%	6 (3-22)	18.2%	24 (15-38)	83	23.6%	11 (<0.1-72)	0	0.0%	-
		2014/15	718	84.3%	9 (3-26)	15.7%	3 (2-8)	61	8.5%	14 (<0.1-70)	13	1.8%	3 (0.2-60)
		2015/16	792	84.8%	7 (2-25)	15.2%	3 (2-11)	109	13.8%	5 (<0.1-67)	5	0.6%	3 (0.2-59)
		2016/17	1,027	78.1%	11 (3-24)	21.9%	14 (2-25)	199	19.4%	16 (<0.1-75)	29	2.8%	2 (<0.1-59)
		2017/18	998	81.0%	9 (3-20)	19.0%	11 (3-21)	164	16.4%	12 (<0.1-72)	44	4.4%	4 (<0.1-79)
		2018/19	879	74.4%	13 (4-24)	25.6%	15 (3-28)	113	12.9%	14 (<0.1-80)	28	3.2%	2 (0.2-23)
		**Total**	**4765**	**80.3%**	**10 (3-23)**	**19.7%**	**8 (2-23)**	**729**	**15.3%**	**13 (<0.1-80)**	**119**	**2.5%**	**3 (<0.1-79)**
	**Khabarovsk**	2013/14	77	100.0%	3 (2-5)	0.0%	-	5	6.5%	3 (2-12)	14	18.2%	2 (1-11)
		2014/15	49	100.0%	3 (1-4)	0.0%	-	8	16.3%	1 (0.4-3)	5	10.2%	1 (0.3-2)
		2015/16	57	100.0%	3 (1-4)	0.0%	-	2	3.5%	2 (2-3)	7	12.3%	2 (0.2-3)
		2016/17	90	100.0%	3 (2-8)	0.0%	-	7	7.8%	2 (1-11)	7	7.8%	2 (0.8-7)
		2017/18	92	100.0%	3 (2-8)	0.0%	-	6	6.5%	0.9 (0.7-1)	4	4.3%	3 (1-4)
		2018/19	76	100.0%	3 (1-10)	0.0%	-	3	3.9%	1 (0.5-10)	10	13.2%	2 (0.2-14)
		**Total**	**441**	**100.0%**	**3 (2-7)**	**0.0%**	**-**	**31**	**7.0%**	**2 (0.4-12)**	**47**	**10.7%**	**2 (0.2-14)**
**Russian Federation, 2013/14 to 2018/19**			**17** **551**	**66.6%**	**9 (3-23)**	**33.4%**	**19 (2-47)**	**2376**	**13.5%**	**15 (<0.1-96)**	**769**	**4.4%**	**3 (<0.1-100)**

*In all sites, data was available from January 2014 to June 2014 for the season 2013/14, and from July to June next year for the remaining five seasons (from 2014/15 to 2018/19.

The A(H1N1)pdm09, A(H3N2) and B influenza virus (sub)types accounted for 31.3%, 44.0% and, respectively, 24.7% of influenza-positive specimens (Table S4 in the **Online Supplementary Document**); only three influenza A cases were unsubtyped. Influenza type A viruses caused >50% of influenza cases in all seasons in all cities except in 2016/17 in Lipetsk and in 2014/2015 in Vladivostok. In 2017/2018 in Vladivostok, the A(H1N1)pdm09, A(H3N2) and B influenza viruses caused each approximately one third of all influenza cases (Table S4 in the **Online Supplementary Document**).

### Age distribution of influenza and RSV patients

The median age of influenza cases was 15 years (IQR 4-34 years), varying from 2 years in Khabarovsk to 29 years in St. Petersburg (**Table 1**). A further source of variability was the level care, as the median age was 9 and 27 years in ILI and SARI patients from the primary and secondary care level, respectively. For RSV, the median age was 2 or 3 years in all surveillance sites, with moderate variability between seasons (range 1 to 6 years) and levels of care (4 and 2 years in primary and secondary care level). The age distribution of those infected with the different influenza virus (sub)types and RSV is depicted in Figures S3-S6 in the **Online Supplementary Document**. The proportion of cases aged ≥65 years was 8.9% for influenza and 3.9% for RSV. Elderly influenza and RSV cases were seen more often in the sites with a larger proportion of specimens contributed by the secondary care level.

Among ILI patients seen at the primary care level, the median age of patients infected with the A(H3N2) influenza virus subtype was 12 years (10^th^ and 90^th^ percentiles: 2 and 43 years), with moderate, yet significant variability between sites (from 8 years in Kaliningrad to 15 years in Chita and Vladivostok; *P* = 0.005). Compared to A(H3N2), the age distribution of A(H1N1)pdm09 patients was skewed towards younger ages (-1 year for the 10^th^ percentile and -5 years for the median, *P* < 0.001 for both), and a parallel, even more pronounced pattern emerged for RSV patients (-1.4 yearsfor the 10^th^ percentile and -8 years for the median, *P* < 0.001) (**Table 2**). Also the median age of influenza B patients was younger (-2 years, *P* = 0.028) than among A(H3N2) patients.

**Table 2:** Comparison of age distribution (10^th^, 50^th^, and 90^th^ percentiles) between virus (sub)types: influenza A(H3N2) (reference), influenza A(H1N1), influenza B, and respiratory syncytial virus (RSV). Simultaneous quantile regression stratified by care level (primary vs secondary) and adjusted by surveillance site

Virus	10^th^ percentile				50^th^ percentile (median)				90^th^ percentile			
Difference (years)*	**95%CI**		***P*-value**	Difference (years)*	**95% CI**		***P*-value**	Difference (years)*	**95% CI**		***P*-value**
**Primary care (patients with ILI)**												
**A(H3N2)**	2 y (ref)				12 y (ref)				43 y (ref)			
**A(H1N1)**	-1	-1.5	-0.5	0.001	-5	-7.0	-3.0	<0.001	0	-9.0	9.0	1.000
**B**	-0.4	-1.5	0.7	0.447	-2	-3.8	-0.2	0.028	-6	-15.1	3.1	0.194
**RSV**	-1.4	-2.0	-0.8	<0.001	-8	-9.7	-6.3	<0.001	-5	-15.5	5.5	0.350
**Secondary care (patients with SARI)**												
**A(H3N2)**	1 y (ref)				26 y (ref)				77 y (ref)			
**A(H1N1)**	0.3	-0.5	1.1	0.474	3	-0.5	6.5	0.095	-6	-12.4	0.4	0.066
**B**	1	-0.2	2.1	0.108	1	-4.6	6.6	0.725	-1	-7.9	5.9	0.775
**RSV**	-0.96	-1.6	-0.3	0.005	-21	-25.8	-16.2	<0.001	-16	-24.9	-7.1	<0.001

ILI – influenza -like illness, SARI – severe acute respiratory infection, CI – confidence interval

*Difference (in years) of the 10^th^, 50^th^ (median), or 90^th^ percentile of the age distribution of patients infected with that virus (influenza A(H1N1), influenza B, or RSV) compared to the age distribution of patients infected with the influenza A(H3N2) virus subtype.

Influenza A(H3N2)-positive SARI patients seen in secondary care had a median age of 26 years (10^th^ and 90^th^ percentile: 1 and 77 years), with substantial variability by site (from 18 years in Vladivostok to 45 years in Chita; *P* < 0.001). There were moderate, non-significant differences in the age distribution of SARI patients infected with the different influenza (sub)types, while RSV patients were much younger (-0.96, -21 and -16 years for the 10^th^, 50^th^ and 90^th^ percentile) compared to A(H3N2) patients (*P* < 0.001) (**Table 2**).

### Seasonality of influenza and RSV epidemics

**Figure 1:**
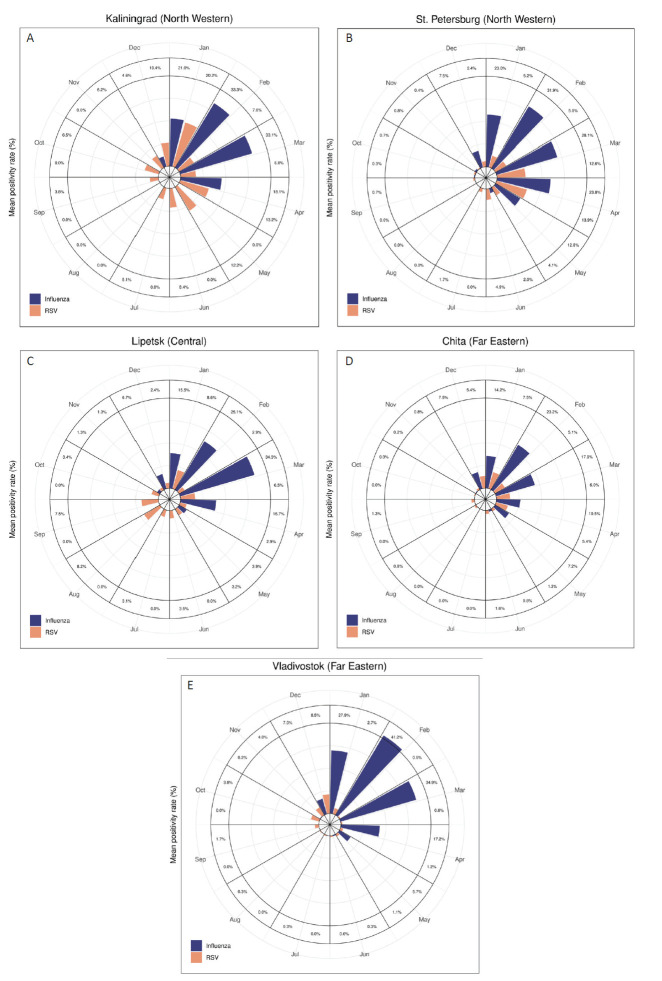
Monthly average positivity rate (%) for influenza and respiratory syncytial virus (RSV) in the surveillance sites of Kaliningrad. (Panel A), St. Petersburg (Panel B), Lipetsk (Panel C), Chita (Panel D), and Vladivostok (panel E). Russia, January 2014 to April 2019.

Data from Khabarovsk were not included in seasonality analyses because of low numbers of reported cases. In the remaining surveillance sites, influenza generally showed a clear seasonality **(****Figure 1**), with the monthly average positivity rate exceeding 20% only in winter months (January to March; the only exception was 23.8% in April in St. Petersburg) (**Figure 1**, Panel B, and Table S5 in the **Online Supplementary Document**). RSV epidemiology resembled that of influenza, with low positivity rate in summer, in St. Petersburg, Chita and Vladivostok (in the latter city, RSV epidemics seemed to precede those of influenza). Instead, RSV cases were distributed with a less distinct seasonal pattern in the other two surveillance sites, with peak or near peak values observed in spring (April-May in Kaliningrad) and late summer (August-September in Lipetsk) (**Figure 1**, Panels A and C, and Table S5 in the **Online Supplementary Document**). Of note, the average month of influenza and RSV epidemic peak differed even between cities located in the same region (eg, Chita and Vladivostok) (**Figure 1**, Panels D and E, and Table S5 in the **Online Supplementary Document**). In addition, there was a substantial season-to-season variability in the timing of the peak of RSV epidemics, at least in some cities (eg, Lipetsk) (Figure S2 in the **Online Supplementary Document**).

The above findings were confirmed when examining the site-specific OR comparing the likelihood of a respiratory specimen testing positive for influenza or RSV in each month compared to the peak month (ie, the month with the highest average positivity rate for that virus in that city) (Figures S7 and S8 and Table S6 in the **Online Supplementary Document**). For influenza, the month with the highest odds of an influenza-positive test was February in all sites except Lipetsk (in March). The OR of an influenza-positive test (compared to the peak month) was below 0.30 from May to December in all sites, and there were a maximum of two months (in Kaliningrad and St. Petersburg) where the odds for an influenza-positive test was not significantly lower than in the peak month.

For RSV, the peak month varied between January (Kaliningrad and Chita), March (St. Petersburg), August (Lipetsk), and December (Vladivostok). Moreover, the number of months in which the odds of a RSV-positive specimen did not significantly differ from that of the peak month was three in Lipetsk (January, May, and September), and up to four in Chita (February-April and December).

## DISCUSSION

We conducted a comparative analysis of the epidemiology of influenza and RSV in Russia using primary- and secondary care-based respiratory virus sentinel surveillance data from six cities between 2014 and 2019. Altogether, 17 551 respiratory specimens were collected, of which 13.5% tested positive for influenza and 4.4% for RSV. On average (ie, by analysing the data from all the cities and seasons together), the median age was higher for influenza virus- than RSV-infected individuals; significant differences in the age distribution of individuals infected with different influenza virus (sub)types emerged in primary care only. The age distribution of those seen in secondary care was shifted towards older ages (for influenza) or younger ages (for RSV) compared to primary care. Influenza epidemics showed a clear seasonality in all surveillance sites, with the peak typically taking place in February or March. In contrast, RSV seasonality showed more variability and was less sharply defined in Kaliningrad and Lipetsk, where RSV was detected year-round.

All influenza virus (sub)types affect people of all ages and, while the attack rates, severity and patients’ age distribution vary from year to year depending on which virus strains are circulating, it is known that the different virus (sub)types typically tend to be reported more frequently in certain age groups [17]. The most important determinants of the severity of influenza illness are a person’s age, comorbidities, and pre-existing immune status; not unsurprisingly, the age distribution of SARI patients was therefore similar for the different influenza virus (sub)types. The modest proportion of cases aged ≥65 years (8.9%) could be explained by the low propensity of elderly Russians suffering from respiratory illnesses to seek medical help. This also helps account for the extremely low proportion of RSV-positive elderly in our study (3.9%), unlike what was reported in several other countries [18-20]. Regarding the age distribution of RSV, it should be noted that a large proportion of young children (especially those aged less than 6 months) infected with RSV do not have fever, and may therefore by missed in surveillance systems that adopt WHO case definitions of ILI and SARI, which include fever [21]. As a consequence, the real age distribution of RSV patients is likely to be even more skewed to the right (compared to influenza patients) than observed in our sample.

We found a clear pattern of annual seasonal epidemics for influenza but much less so for RSV. Even where RSV showed more defined seasonality, the peak month mostly did not occur around the time of that of influenza. The reason behind this dual source of variability (between viruses and across sites) in the timing of RSV epidemics is unclear. Influenza epidemics in Russia may spread eastwards in the West of the country, and westwards in the East of the country, depending on the season [22]. This would entail that the eastern and western regions of the country may be affected by influenza epidemics at different times, however, this pattern did not emerge in our study. This could be explained by Russia having a sparse population scattered over a vast territory, with the capital city (Moscow) representing a central transmission hub, where people from the whole country come into contact, thus accelerating the spread of the epidemics.

Our results have substantial public health implications. The typical timing of influenza epidemics in Russia seems to be fairly aligned throughout the country, so there is no need to establish region-specific influenza vaccination strategies, unlike vast countries that span a wide latitude range [23-25]. However, the average influenza positivity rate was above 10% from January to April in all cities, and as late as May in St. Petersburg. Given waning vaccine-induced immunity [26,27], persons vaccinated early during the immunization campaign may lack protection at the tail end of the epidemic season, thus it might be beneficial to shift the start of the vaccination programme to a later date. For instance, in the USA it is recommended to be vaccinated by the end of October, yet vaccines administered as late as January might be still beneficial if the peak of influenza activity falls in February-March, which occurs in over half of the seasons [28]. Regarding the administration of PVZ prophylaxis to eligible infants (premature infants and children with bronchopulmonary dysplasia or hemodynamically significant congenital heart disease [29]), the situation is complex as some cities in Russia do not show short periods of RSV circulation, unlike in most Western Europe [30]. PVZ is usually given before the RSV start and then once a month as long as RSV circulates, ie, generally five months in most of Western Europe [31]. In those regions of Russia where RSV circulates for extended periods throughout the year, PVZ prophylaxis might need to be administered longer if one wants to ensure full protection. Considering, however, that PVZ is an expensive drug [32], the question of regional RSV seasonality and the delivery of PVZ prophylaxis needs to be carefully considered. Since 2015 in Russia, PVZ has been included in the list of vital medicines for infants in the first year of age at risk for severe RSV infection. In our analysis, RSV had a positivity rate three times lower than influenza, yet it is the major cause of SARI in infants and young children, many of whom are currently not included in the risk groups indicated in the MoH guidelines and thus not eligible for PVZ use. This further highlights the need of ongoing RSV surveillance and development of effective RSV vaccines.

To our knowledge, this is the first comparative description of the epidemiology of influenza and RSV in Russia, and is one of a small number of studies to assess epidemiology of both viruses in a harmonised manner [33]. The study database encompassed surveillance data from multiple sites distributed from the pacific coast to the extreme west of the country, and individual information was available on the patient’s age, causative agent, and level of care. Our study had a number of limitations, the most important of which are the sparse geographic representation and the relatively small sample size (particularly in some surveillance sites) of data. In particular, only six out of the ten cities included in the surveillance system in Russia could be included in the analysis. Because of the limited geographical representativeness, we were unable to calculate an estimate of influenza and RSV disease burden (mortality, hospitalizations, or total illness) in Russia. The proportion of patients seen at the primary and secondary care level varied between surveillance sites, which may undermine their comparability. Furthermore, individuals infected with influenza B or RSV were not subtyped into Victoria/Yamagata lineages or, respectively, A/B virus types, while it is known that this can affect the age distribution [34]. Also, influenza and RSV co-infections were not studied, although they may be common when the two viruses co-circulate and may have clinical implications [35]. As already mentioned, the comparison of age distribution of influenza and RSV patients may be biased by a number of factors, including the inability of an ILI/SARI-based surveillance system to detect the proportion of very young (<2 years of age) RSV patients who do not have fever, and the fact that the propensity to seek medical care may differ by patients’ age (and is much lower in Russia for the adults and especially the elderly than for children) and also by site. Thus, what we see in our study is the age distribution of medically-attended influenza and RSV cases at each sentinel site, rather than the actual age distribution of people infected with influenza or RSV in the whole country. Finally, limitations in the number of cases forced us to use the month, instead of the week, as the unit of analysis of spatiotemporal epidemiology of epidemics. The combination of the rapid spread of epidemics and of the suboptimal temporal resolution of our analysis may have contributed to our inability to detect a geographic gradient in the spread of influenza and RSV epidemics in Russia.

## CONCLUSIONS

We found that the epidemiology of influenza and RSV in Russia differed in terms of the timing of epidemics in addition to the typical age distribution of those who are infected. Our results represent an important reference on which to base public health decisions aimed at containing the burden of diseases of these two respiratory infections in Russia, in particular, the optimal time to start the implementation of influenza vaccination campaigns and the timing and duration of RSV prophylaxis.

## Additional material


Online Supplementary Document
