# Supplementary material for: A comparative analysis of the epidemiology of influenza and respiratory syncytial virus in Russia, 2013/14 to 2018/19
Source: J Glob Health. 2022 Feb 5;12:04009. doi: 10.7189/jogh.12.04009 (PMC8818296; doi:10.7189/jogh.12.04009)
Supplement: Online Supplementary Document [file jogh-12-04009-s001.pdf]

Table S1. Number of polyclinics (primary care level) and infectious disease hospitals (secondary care level) included in each respiratory viral surveillance site.

City	Primary care level	Secondary care level
**North-Western Federal District**		
Kaliningrad	1 adult polyclinic<br>1 children polyclinic	1 adult infectious disease hospital<br>1 children infectious disease hospital
Saint Petersburg	1 polyclinic (children and adults)	1 adult infectious disease hospital<br>1 children infectious disease hospital
**Central Federal District**		
Lipetsk	1 adult polyclinic<br>1 children polyclinic	1 infectious disease hospital (children and adults)
**Far-Eastern Federal District**		
Chita	1 adult polyclinic<br>1 children polyclinic	2 adult infectious disease hospitals<br>2 children infectious disease hospitals
Vladivostok	1 adult polyclinic<br>1 children polyclinic	1 adult infectious disease hospital<br>1 children infectious disease hospital
Khabarovsk	1 adult polyclinic<br>1 children polyclinic	none

Table S2. Positivity rate for influenza and RSV stratified by surveillance site, season, and level of care.

Federal District	Site	Season [(a)]	Positivity rate influenza (%)		Positivity rate RSV (%)	
			Primary care	Secondary care	Primary care	Secondary care
North Western	Kaliningrad	2013/14	-	-	-	-
		2014/15	-	-	-	-
		2015/16	-	-	-	-
		2016/17	24.7%	40.0%	7.7%	15.0%
		2017/18	-	-	-	-
		2018/19	27.2%	6.5%	14.2%	22.9%
	St. Petersburg	2013/14	2.0%	24.2%	3.0%	10.7%
		2014/15	8.3%	23.3%	2.0%	4.1%
		2015/16	17.9%	21.3%	2.2%	7.7%
		2016/17	10.1%	15.4%	0.0%	5.2%
		2017/18	6.6%	21.3%	2.3%	4.7%
		2018/19	5.1%	10.1%	3.9%	6.5%
Central	Lipetsk	2013/14	26.1%	14.9%	0.4%	0.0%
		2014/15	17.1%	21.4%	2.2%	0.0%
		2015/16	-	-	-	-
		2016/17	-	-	-	-
		2017/18	6.2%	3.1%	13.4%	0.0%
		2018/19	-	-	-	-
Far Eastern	Chita	2013/14	15.5%	19.5%	5.3%	6.8%
		2014/15	9.1%	11.7%	1.8%	4.2%
		2015/16	5.9%	8.5%	2.6%	3.7%
		2016/17	9.0%	12.4%	1.4%	3.2%
		2017/18	6.2%	6.4%	3.9%	5.3%
		2018/19	8.7%	7.8%	3.5%	6.8%
	Vladivostok	2013/14	17.8%	50.0%	0.0%	0.0%
		2014/15	7.3%	15.0%	1.5%	3.5%
		2015/16	12.8%	19.2%	0.3%	2.5%
		2016/17	15.8%	32.0%	1.9%	6.2%
		2017/18	15.2%	21.6%	3.5%	8.4%
		2018/19	9.6%	15.4%	2.6%	4.9%

[(a)] In all sites, data was available from January 2014 to June 2014 for the season 2013/14, and from July to June next year for the remaining five seasons (from 2014/15 to 2018/19). Only seasons with at least 50 samples tested at both levels of care were included.

Table S3. Positivity rate for respiratory viruses other than influenza and respiratory syncytial virus by surveillance site and season. Samples from ILI and SARI patients were merged before calculating virus-specific positivity rates.

Surveillance site	Season	Parainfluenza viruses	Adenoviruses	Seasonal coronaviruses	Human metapneumovirus	Bocavirus	Rhinoviruses
**Kaliningrad**	2013/14	8.2%	7.7%	0.0%	0.0%	0.5%	1.8%
	2014/15	12.3%	12.3%	0.0%	0.0%	0.0%	4.5%
	2015/16	12.0%	11.2%	0.4%	1.2%	0.4%	4.4%
	2016/17	5.4%	5.4%	0.0%	0.7%	0.7%	1.8%
	2017/18	13.2%	12.9%	0.0%	0.0%	0.0%	4.3%
	2018/19	8.2%	12.8%	0.0%	0.5%	1.0%	3.6%
	**Total**	**9.6%**	**10.5%**	**0.1%**	**0.4%**	**0.5%**	**3.4%**
**St. Petersburg**	2013/14	6.8%	0.8%	0.0%	0.0%	0.2%	4.2%
	2014/15	4.1%	4.1%	0.0%	0.0%	0.1%	1.2%
	2015/16	4.5%	2.4%	1.3%	0.1%	1.0%	5.7%
	2016/17	6.1%	2.7%	2.0%	2.4%	1.2%	20.2%
	2017/18	5.9%	2.8%	2.3%	1.9%	1.9%	14.9%
	2018/19	6.1%	1.5%	3.9%	4.4%	2.5%	14.6%
	**Total**	**5.5%**	**2.5%**	**1.6%**	**1.4%**	**1.1%**	**10.1%**
**Lipetsk**	2013/14	17.9%	9.2%	0.0%	0.0%	0.2%	0.2%
	2014/15	12.3%	7.2%	0.0%	0.0%	0.0%	0.0%
	2015/16	13.1%	10.0%	0.0%	0.0%	0.0%	0.3%
	2016/17	26.8%	14.8%	0.0%	0.0%	0.0%	0.0%
	2017/18	20.8%	10.0%	0.0%	0.0%	0.0%	0.0%
	2018/19	24.5%	19.7%	0.0%	0.0%	0.0%	0.5%

	**Total**	**18.8%**	**11.3%**	**0.0%**	**0.0%**	**0.0%**	**0.1%**
**Chita**	2013/14	5.3%	0.8%	1.0%	2.8%	0.5%	9.0%
	2014/15	2.3%	0.8%	0.0%	0.8%	0.8%	16.3%
	2015/16	4.4%	2.3%	0.7%	0.8%	0.4%	18.0%
	2016/17	3.1%	2.0%	1.8%	2.5%	0.4%	18.3%
	2017/18	2.3%	1.2%	0.8%	1.0%	0.5%	20.1%
	2018/19	2.7%	0.8%	2.6%	1.6%	0.5%	19.8%
	**Total**	**3.4%**	**1.3%**	**1.2%**	**1.6%**	**0.5%**	**16.8%**
**Vladivostok**	2013/14	5.2%	1.9%	0.5%	1.9%	2.4%	9.3%
	2014/15	4.5%	2.3%	0.1%	2.6%	0.8%	13.2%
	2015/16	2.9%	2.3%	0.0%	0.7%	1.2%	14.0%
	2016/17	2.1%	1.7%	0.1%	1.5%	0.5%	13.6%
	2017/18	4.4%	1.1%	0.7%	3.9%	0.7%	13.1%
	2018/19	2.3%	1.3%	0.8%	1.7%	1.2%	10.0%
	**Total**	**3.4%**	**1.7%**	**0.4%**	**2.0%**	**1.1%**	**12.3%**
**Khabarovsk**	2013/14	0.0%	5.7%	0.0%	8.1%	0.8%	25.2%
	2014/15	4.3%	4.3%	0.0%	4.3%	2.2%	13.0%
	2015/16	8.3%	0.0%	1.7%	1.7%	1.7%	30.0%
	2016/17	3.8%	6.3%	0.0%	3.8%	0.0%	1.3%
	2017/18	3.1%	3.1%	0.0%	3.1%	1.0%	19.8%
	2018/19	1.5%	0.0%	1.5%	6.1%	0.0%	18.2%
	**Total**	**3.0%**	**3.6%**	**0.4%**	**4.9%**	**0.8%**	**18.5%**

Table S4. Proportion of influenza cases by virus (sub)type in each site and season.

Federal District	Site	Season *	Influenza			
			No. cases †	A(H1N1)	A(H3N2)	B
North Western	Kaliningrad	2013/14	22	-	-	-
		2014/15	8	-	-	-
		2015/16	26	-	-	-
		2016/17	88	0.0%	58.0%	42.0%
		2017/18	23	-	-	-
		2018/19	69	58.0%	36.2%	5.8%
		**Total**	**236**	**32.6%**	**44.9%**	**22.5%**
	St. Petersburg	2013/14	53	11.3%	86.8%	1.9%
		2014/15	142	4.9%	59.9%	35.2%
		2015/16	123	88.6%	0.8%	10.6%
		2016/17	97	3.1%	71.1%	25.8%
		2017/18	134	6.0%	46.3%	47.8%
		2018/19	53	41.5%	56.6%	1.9%
		**Total**	**602**	**25.7%**	**48.7%**	**25.6%**
Central	Lipetsk	2013/14	88	1.1%	89.8%	9.1%
		2014/15	88	3.4%	60.2%	36.4%
		2015/16	55	100.0%	0.0%	0.0%
		2016/17	78	0.0%	46.2%	53.8%
		2017/18	32	-	-	-
		2018/19	36	-	-	-
		**Total**	**377**	**24.4%**	**53.1%**	**22.5%**
Far Eastern	Chita	2013/14	75	84.0%	1.3%	14.7%
		2014/15	72	0.0%	59.7%	40.3%
		2015/16	55	49.1%	40.0%	10.9%
		2016/17	86	0.0%	58.1%	41.9%
		2017/18	52	7.7%	67.3%	25.0%
		2018/19	60	81.7%	18.3%	0.0%
		**Total**	**400**	**35.8%**	**40.5%**	**23.8%**
	Vladivostok	2013/14	83	65.1%	2.4%	32.5%

		2014/15	111	1.8%	35.1%	63.1%
		2015/16	107	65.4%	29.0%	5.6%
		2016/17	199	0.0%	63.3%	36.7%
		2017/18	164	32.9%	31.7%	35.4%
		2018/19	113	78.8%	19.5%	1.8%
		**Total**	**727**	**37.0%**	**37.4%**	**25.6%**
	**Khabarovsk**	2013/14	5	-	-	-
		2014/15	8	-	-	-
		2015/16	2	-	-	-
		2016/17	7	-	-	-
		2017/18	6	-	-	-
		2018/19	3	-	-	-
		**Total**	**31**	**-**	**-**	**-**

* For all sites, data was available from January 2014 to April 2019.

† The proportion of influenza cases caused by each influenza virus (sub)type was calculated only in seasons with at least 50 reported influenza cases.

Table S5. Monthly average positivity rate for influenza and respiratory syncytial virus (RSV) in each surveillance site in Russia (from January 2014 to April 2019).

District	North Western				Central		Far Eastern			
Site	Kaliningrad		St. Petersburg		Lipetsk		Chita		Vladivostok	
Virus	Influenza	RSV	Influenza	RSV	Influenza	RSV	Influenza	RSV	Influenza	RSV
**Month**	**Monthly average positivity rate (%)**									
Jan	21.0%	**20.2%**	23.0%	5.2%	15.5%	**8.6%**	14.2%	**7.5%**	27.9%	2.7%
Feb	**33.3%**	7.6%	**31.9%**	5.5%	25.1%	2.9%	**23.2%**	5.1%	**41.2%**	0.5%
Mar	33.1%	6.8%	28.1%	12.6%	**34.3%**	6.5%	17.9%	6.0%	34.9%	0.6%
Apr	18.1%	13.2%	23.8%	**13.9%**	15.7%	2.9%	10.5%	5.4%	17.2%	1.2%
May	0.0%	12.2%	12.8%	4.1%	3.9%	3.2%	7.2%	1.3%	5.7%	1.1%
Jun	0.0%	8.4%	2.0%	4.9%	0.0%	3.5%	0.8%	1.6%	0.3%	0.6%
Jul	0.0%	5.1%	0.0%	1.7%	0.0%	3.1%	0.0%	0.0%	0.0%	0.3%
Aug	0.0%	0.0%	0.0%	0.0%	0.0%	8.2%	0.0%	0.8%	0.0%	0.3%
Sep	0.0%	3.8%	0.0%	0.7%	0.0%	7.5%	0.0%	1.3%	0.0%	1.7%
Oct	0.0%	6.5%	0.3%	0.7%	0.0%	3.4%	0.0%	0.3%	0.0%	3.8%
Nov	0.0%	6.2%	0.8%	0.4%	1.3%	1.3%	0.2%	0.8%	0.2%	4.0%
Dec	4.6%	10.4%	7.5%	2.4%	6.7%	2.4%	7.5%	5.4%	7.0%	**8.5%**

Table S6. Seasonality of influenza epidemics in each surveillance site in Russia (January 2014 to April 2019). Odds ratio (OR) with corresponding 95% confidence intervals for testing positive for influenza or respiratory syncytial virus (RSV) in each month compared to the month with the highest OR for a positive test in each surveillance site (reference, in bold).

District	North Western						Central			Far Eastern					
Site	Kaliningrad			St. Petersburg			Lipetsk			Chita			Vladivostok		
Month	OR	95% CI	p-value	OR	95% CI	p-value	OR	95% CI	p-value	OR	95% CI	p-value	OR	95% CI	p-value
**Influenza**															
Jan	0.69	0.44-1.06	0.093	0.62	0.46-0.83	0.001	0.43	0.29-0.63	<0.001	0.55	0.40-0.77	<0.001	0.58	0.45-0.75	<0.001
Feb	**1.00**			**1.00**			0.59	0.44-0.80	<0.001	**1.00**			**1.00**		
Mar	0.82	0.54-1.23	0.327	0.82	0.63-1.07	0.141	**1.00**			0.74	0.55-0.99	0.040	0.74	0.58-0.94	0.014
Apr	0.35	0.21-0.60	<0.001	0.79	0.59-1.04	0.098	0.31	0.22-0.45	<0.001	0.40	0.28-0.56	<0.001	0.30	0.22-0.39	<0.001
May	-	-	-	0.25	0.16-0.40	<0.001	0.06	0.02-0.13	<0.001	0.26	0.15-0.44	<0.001	0.07	0.04-0.13	<0.001
Jun	-	-	-	0.04	0.01-0.10	<0.001	-	-	-	0.02	0.003-0.15	<0.001	0.01	0.001-0.04	<0.001
Jul	-	-	-	-	-	-	-	-	-	-	-	-	-	-	-
Aug	-	-	-	-	-	-	-	-	-	-	-	-	-	-	-
Sep	-	-	-	-	-	-	-	-	-	-	-	-	-	-	-
Oct	-	-	-	0.01	0.001-0.04	<0.001	-	-	-	-	-	-	-	-	-
Nov	-	-	-	0.01	0.003-0.05	<0.001	0.02	0.006-0.06	<0.001	0.01	0.001-0.06	<0.001	0.01	0.001-0.02	<0.001
Dec	0.12	0.06-0.22	<0.001	0.15	0.10-0.25	<0.001	0.12	0.08-0.20	<0.001	0.25	0.17-0.38	<0.001	0.14	0.09-0.19	<0.001
**RSV**															
Jan	**1.00**			0.43	0.24-0.75	0.003	0.60	0.29-1.25	0.174	**1.00**			0.33	0.19-0.59	<0.001
Feb	0.30	0.16-0.57	<0.001	0.45	0.28-0.74	0.002	0.30	0.14-0.63	0.002	0.72	0.42-1.21	0.214	0.07	0.03-0.20	<0.001
Mar	0.23	0.11-0.46	<0.001	0.97	0.63-1.50	0.898	0.41	0.20-0.82	0.012	0.79	0.48-1.31	0.367	0.07	0.03-0.20	<0.001
Apr	0.54	0.28-1.03	0.063	**1.00**			0.34	0.16-0.74	0.006	0.71	0.41-1.20	0.201	0.14	0.06-0.31	<0.001
May	0.46	0.18-1.18	0.107	0.32	0.15-0.70	0.004	0.45	0.19-1.05	0.066	0.18	0.05-0.59	0.005	0.13	0.05-0.37	<0.001
Jun	0.39	0.16-0.92	0.032	0.36	0.17-0.76	0.007	0.36	0.14-0.96	0.041	0.26	0.08-0.86	0.027	0.04	0.01-0.26	0.001
Jul	0.17	0.05-0.56	0.004	0.10	0.03-0.34	<0.001	0.34	0.13-0.90	0.030	-	-	-	0.04	0.01-0.26	0.001
Aug	-	-	-	-	-	-	**1.00**			0.08	0.01-0.60	0.014	0.04	0.01-0.28	0.001

Sep	0.14	0.04-0.48	0.002	0.06	0.01-0.24	<0.001	0.71	0.34-1.47	0.354	0.16	0.04-0.67	0.012	0.22	0.09-0.53	<0.001
Oct	0.26	0.13-0.56	<0.001	0.05	0.01-0.22	<0.001	0.30	0.13-0.69	0.005	0.04	0.005-0.26	0.001	0.41	0.22-0.77	0.005
Nov	0.39	0.20-0.77	0.007	0.03	0.004-0.21	<0.001	0.10	0.03-0.35	<0.001	0.10	0.03-0.34	<0.001	0.38	0.21-0.69	0.001
Dec	0.54	0.30-0.97	0.039	0.23	0.11-0.51	<0.001	0.28	0.12-0.64	0.003	0.71	0.41-1.23	0.222	**1.00**		

Figure S1. Map of the Russian Federation showing the geographical distribution of the cities included in the Sentinel Surveillance System. Data from cities in dark blue areas were included in the present analysis, while those in light blue area were not included (see text for details).

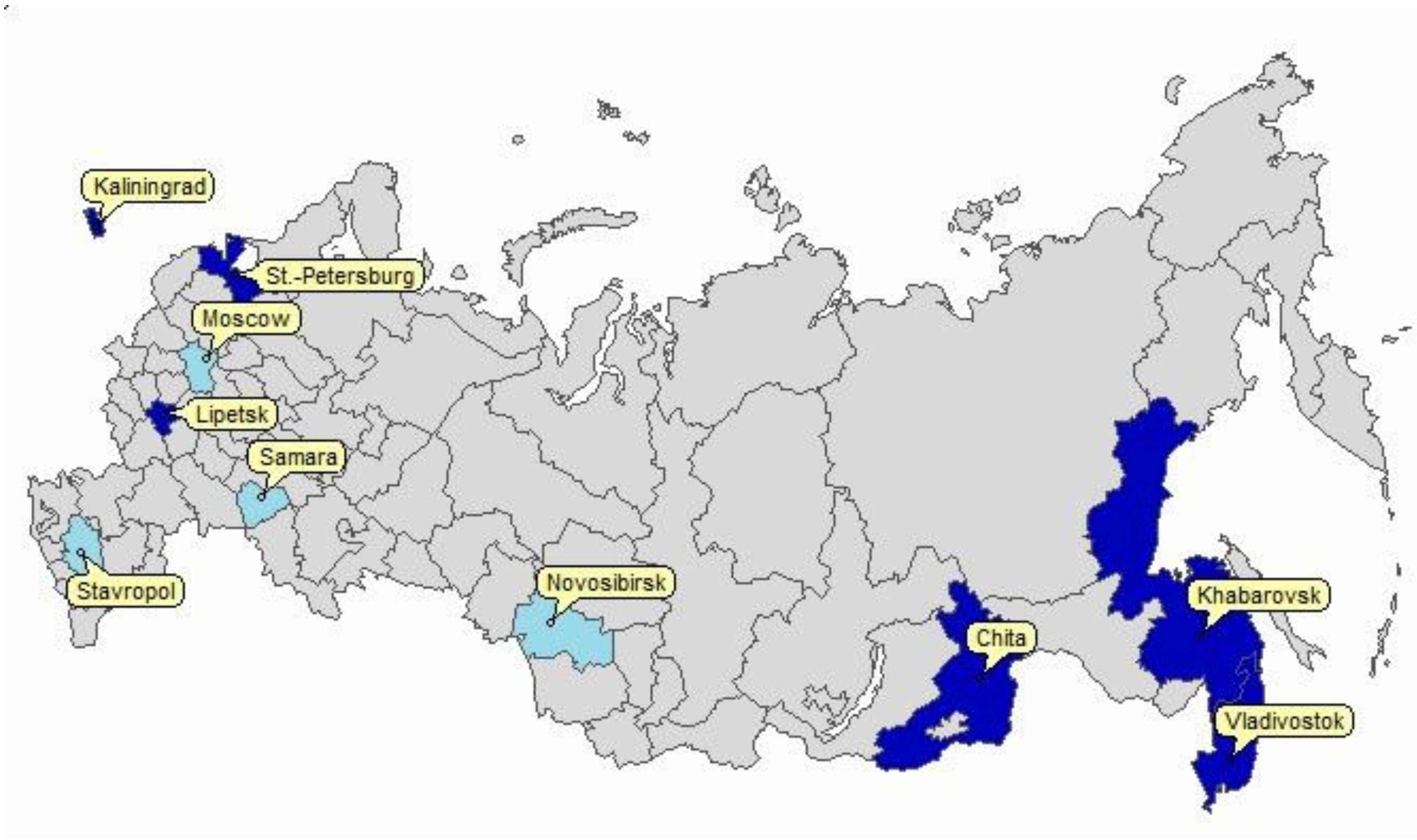

Figure S2. Monthly number of respiratory specimens (grey) and of those testing positive for influenza virus (red) or respiratory syncytial virus (blue) by surveillance site: Kaliningrad (panel a), St. Petersburg (panel b), Lipetsk (panel c), Chita (panel d), Vladivostok (panel e), and Khabarovsk (panel f). Russia, January 2014 to April 2019. Samples from ILI and SARI patients were merged before calculating virus-specific positivity rates.

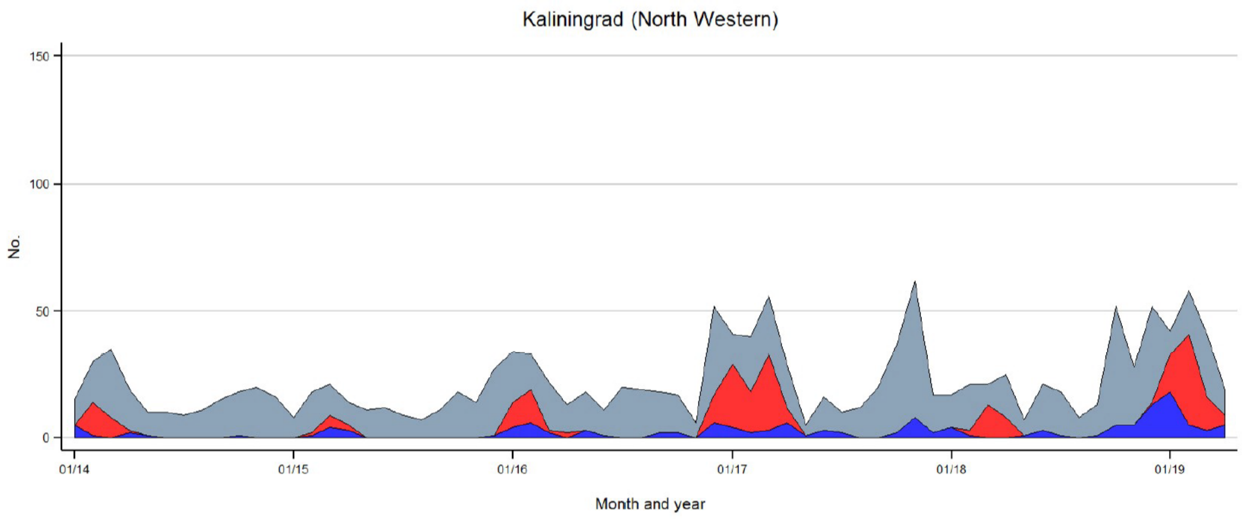

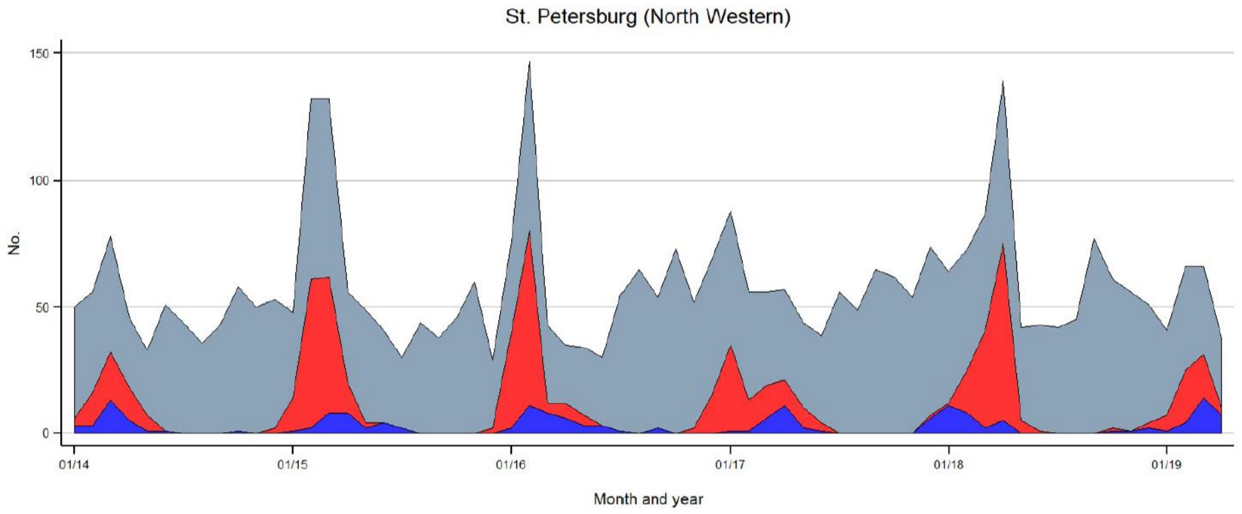

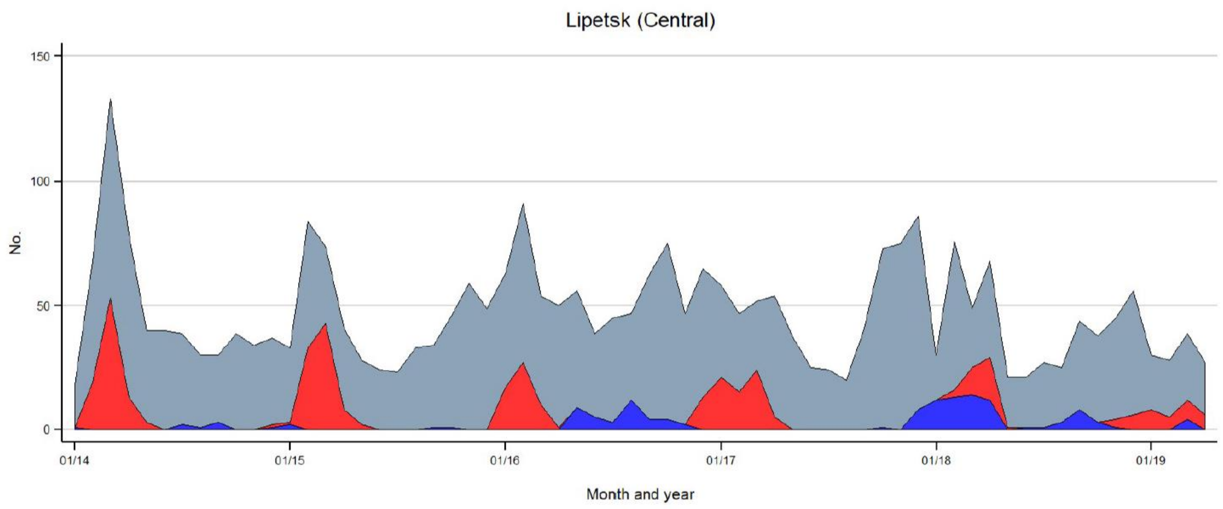
Lipetsk (Central)
150
100
50
0
No.
01/14
01/15
01/16
01/17
01/18
01/19
Month and year

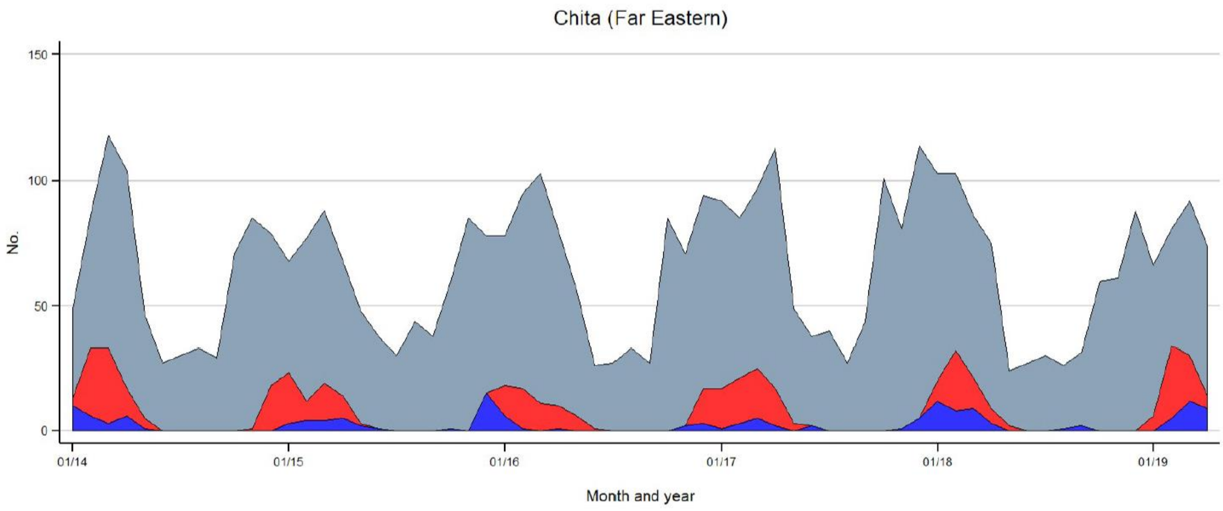
Chita (Far Eastern)
150
100
50
0
No.
01/14
01/15
01/16
01/17
01/18
01/19
Month and year

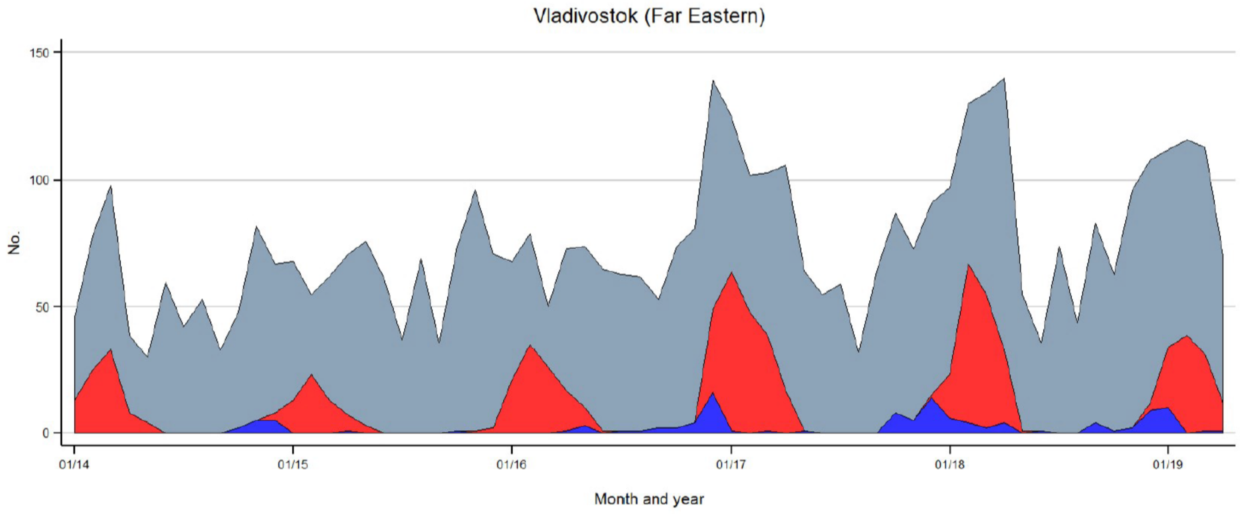
Vladivostok (Far Eastern)
150
100
50
0
No.
01/14
01/15
01/16
01/17
01/18
01/19
Month and year

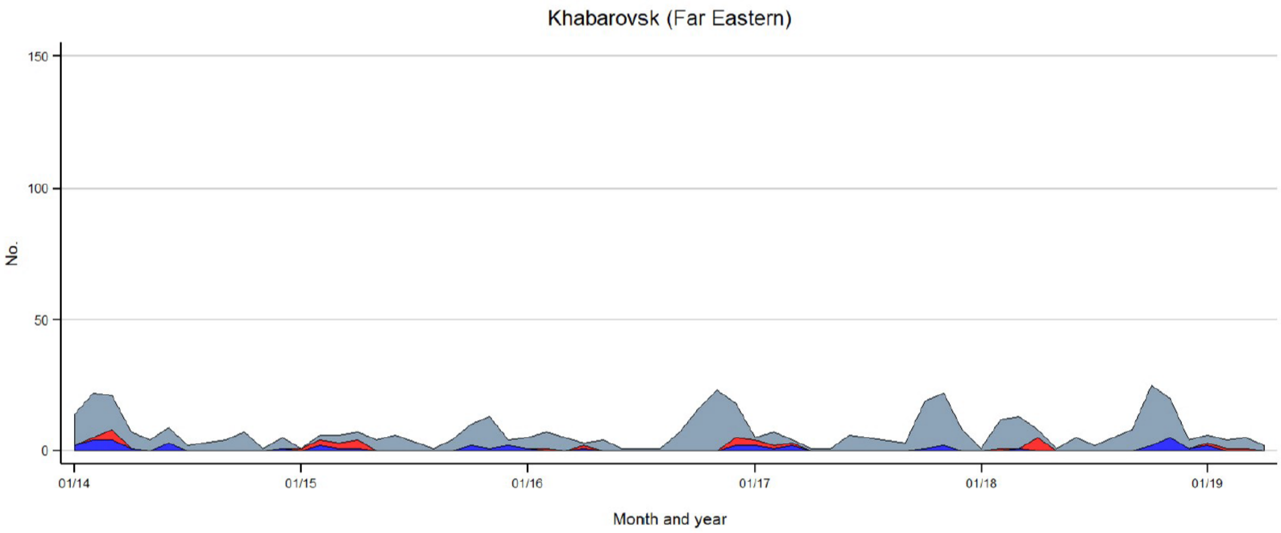
Khabarovsk (Far Eastern)
No.
150
100
50
0
01/14
01/15
01/16
01/17
01/18
01/19
Month and year

Figure S3. Age distribution of patients infected with influenza A(H1N1)pdm09 virus in primary and secondary level of care. Russia, January 2014 to April 2019.

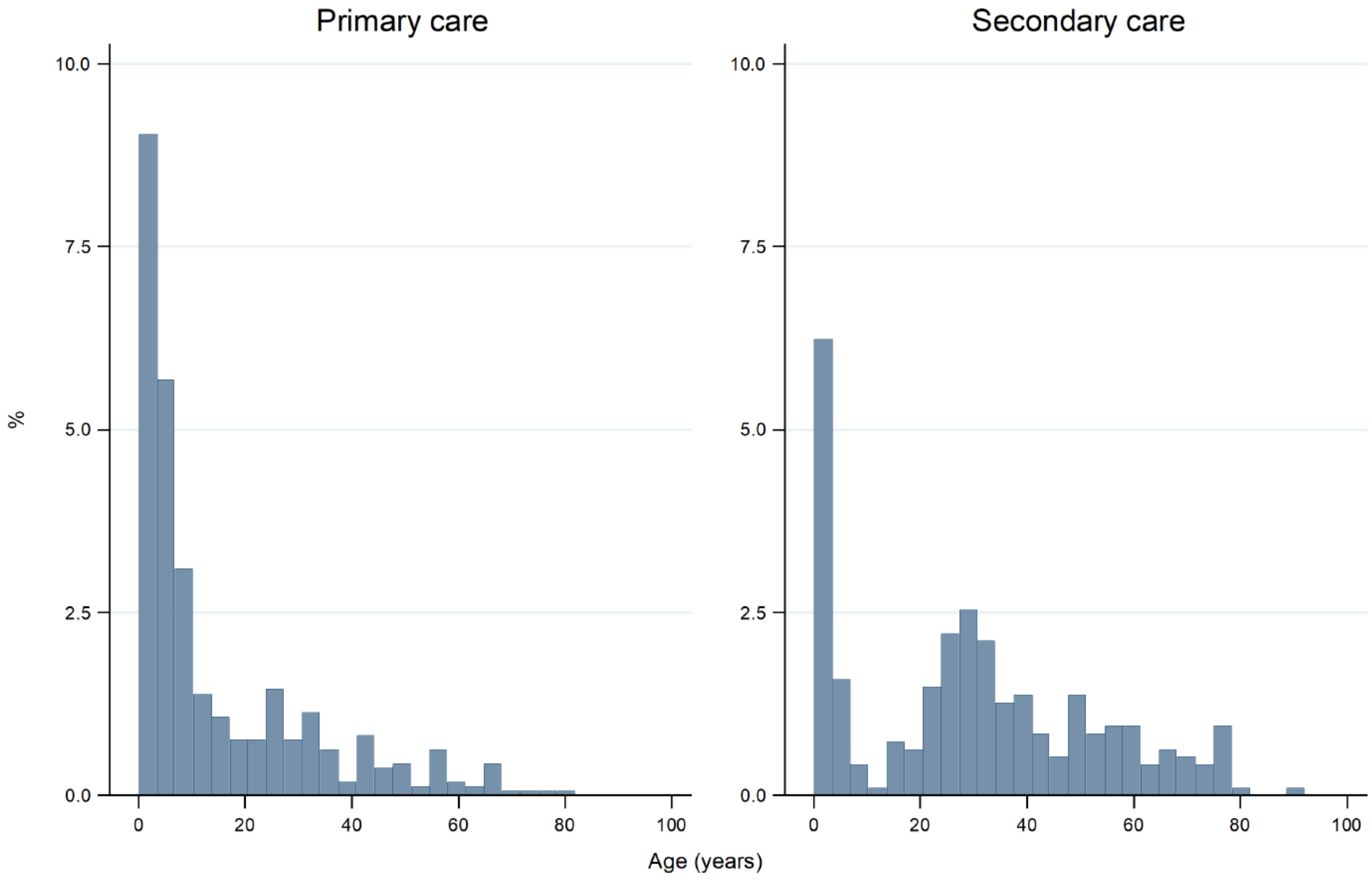

Figure S4. Age distribution of patients infected with influenza A(H3N2) virus in primary and secondary level of care. Russia, January 2014 to April 2019.

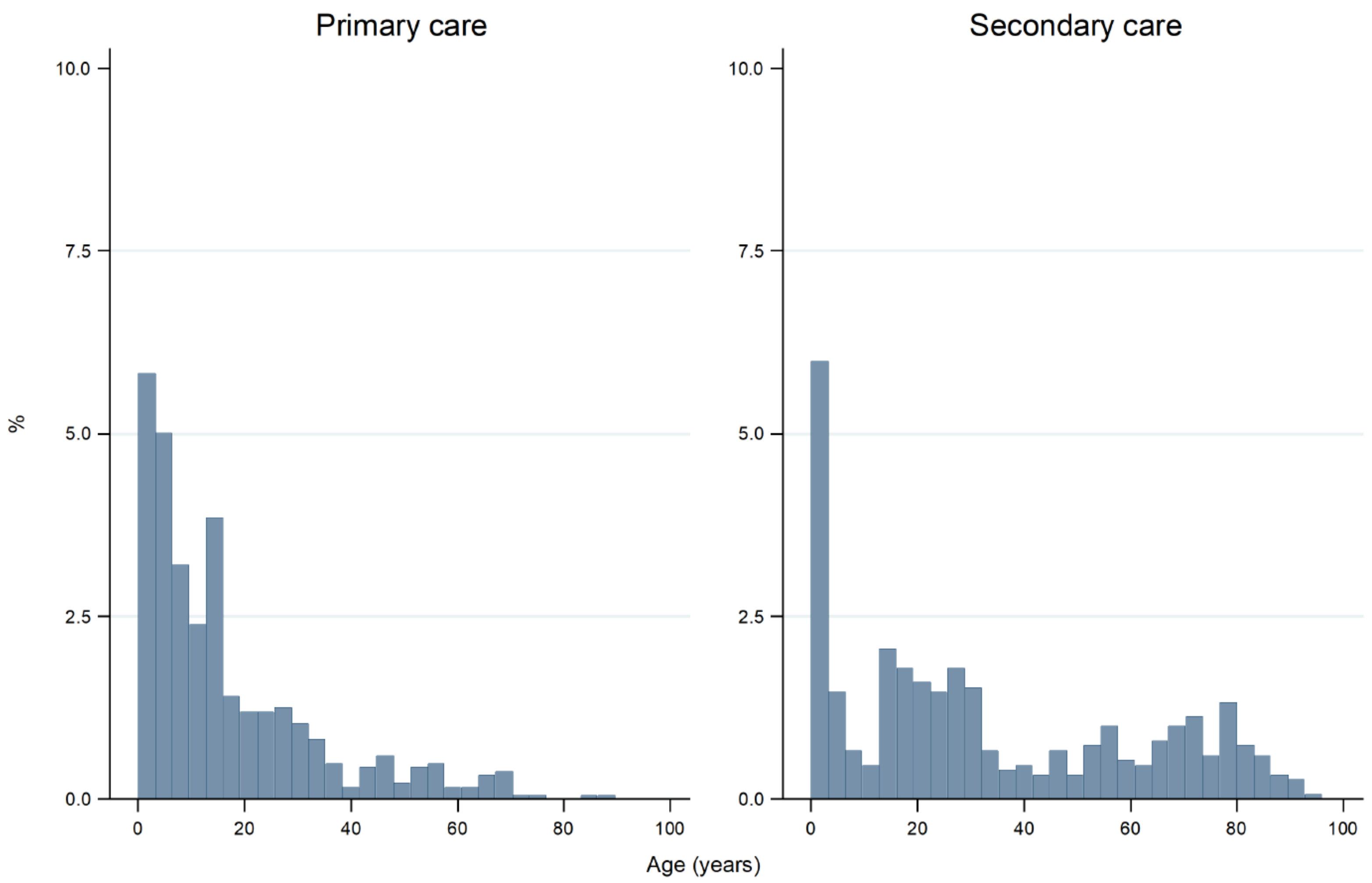

Figure S5. Age distribution of patients infected with influenza B virus in primary and secondary level of care. Russia, January 2014 to April 2019.

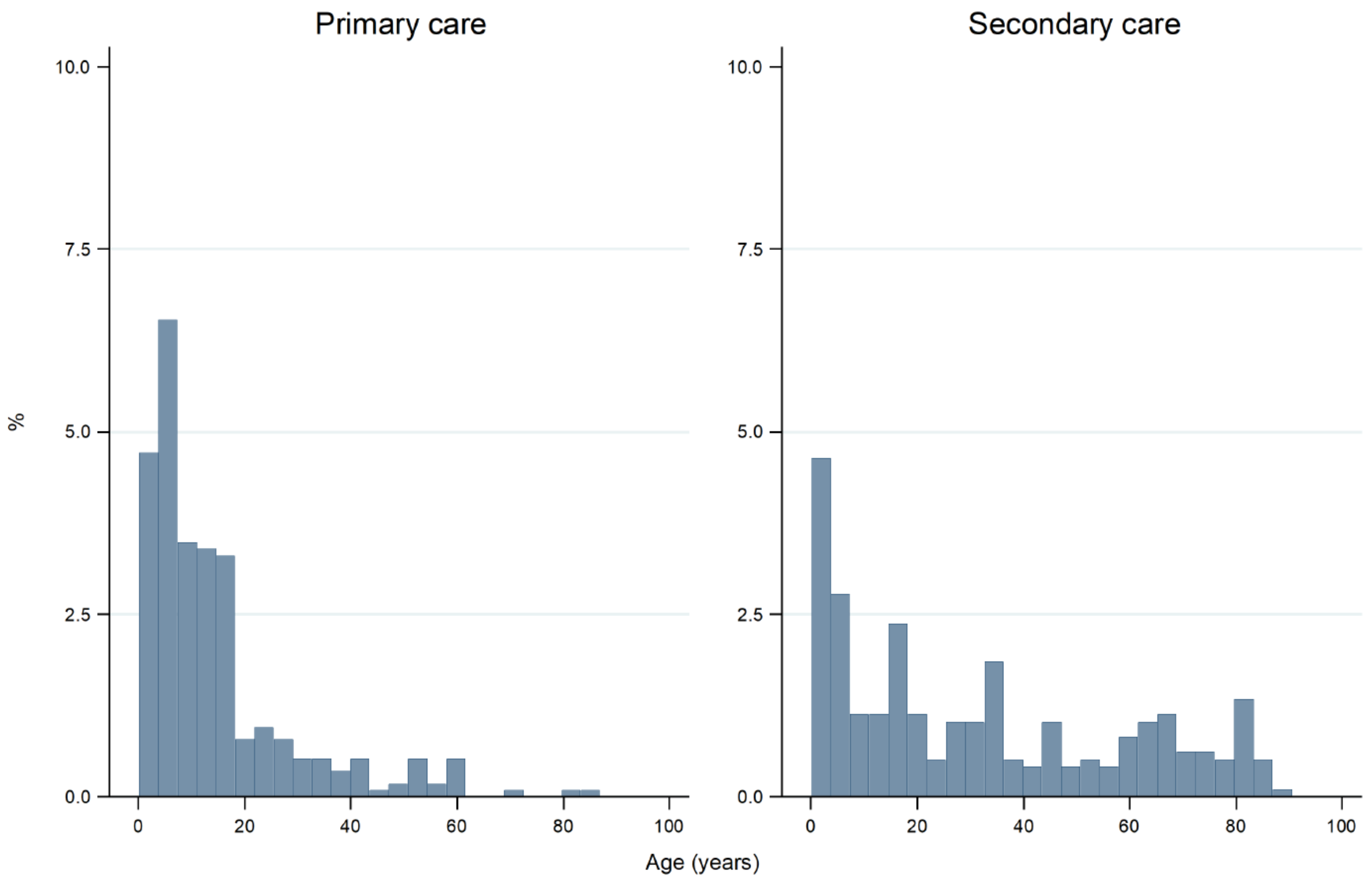

Figure S6. Age distribution of patients infected with respiratory syncytial virus in primary and secondary level of care. Russia, January 2014 to April 2019.

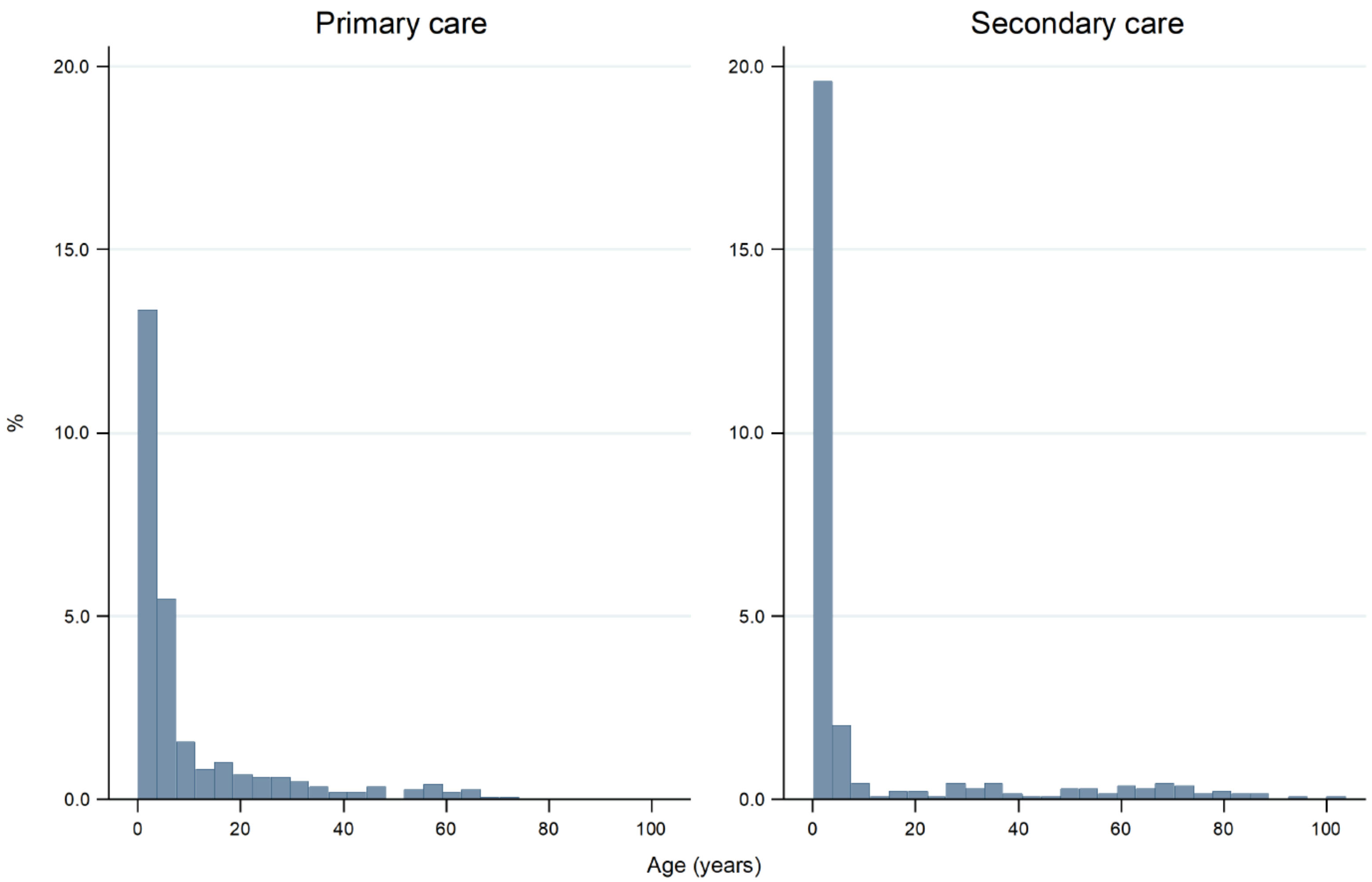

Figure S7. Seasonality of influenza epidemics in each surveillance site in Russia (January 2014 to April 2019): Kaliningrad (panel a), St. Petersburg (panel b), Lipetsk (panel c), Chita (panel d), and Vladivostok (panel e). Odds ratio (OR) with corresponding 95% confidence intervals for having an influenza-positive test in each month compared to the peak month (reference).

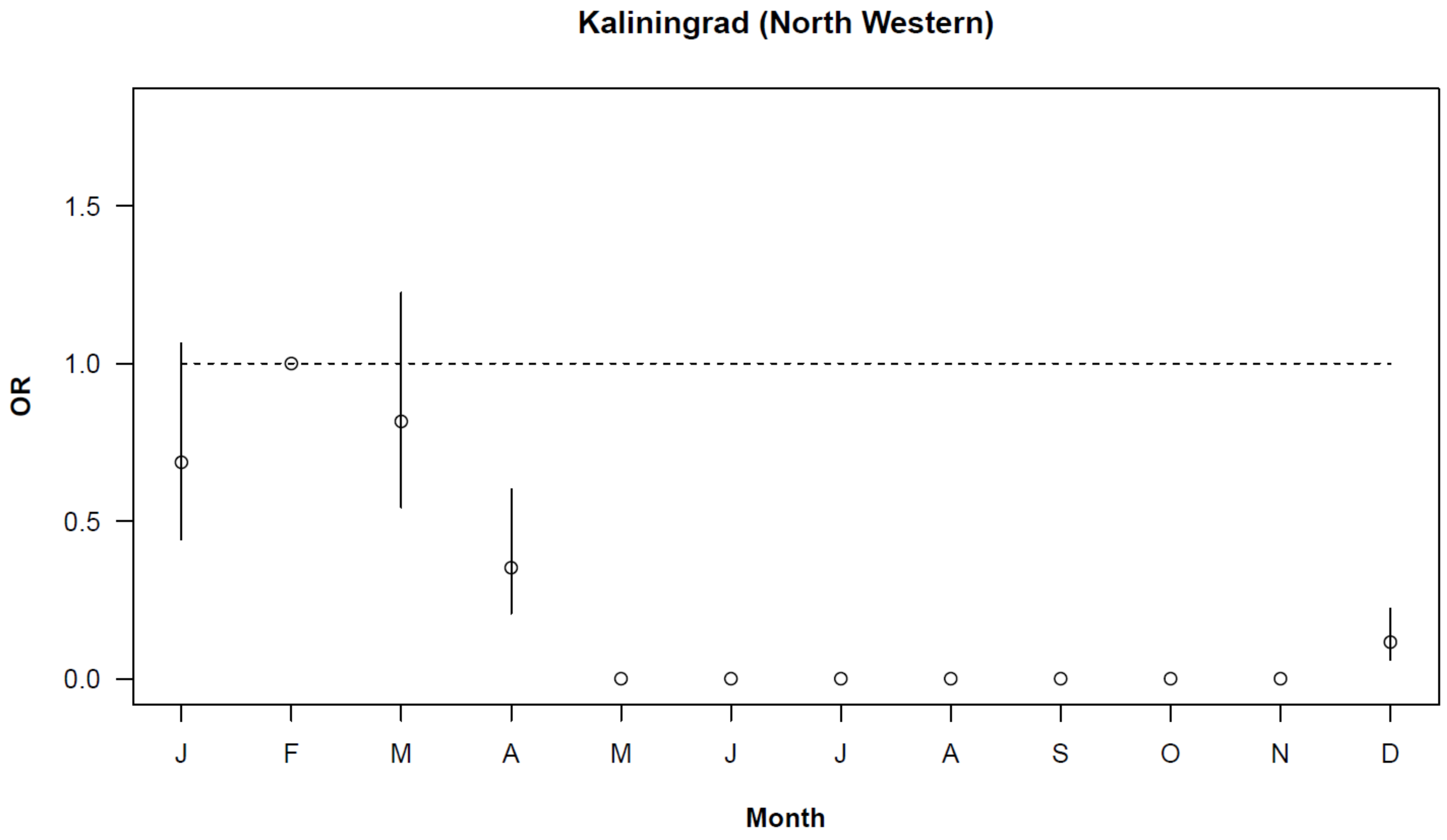

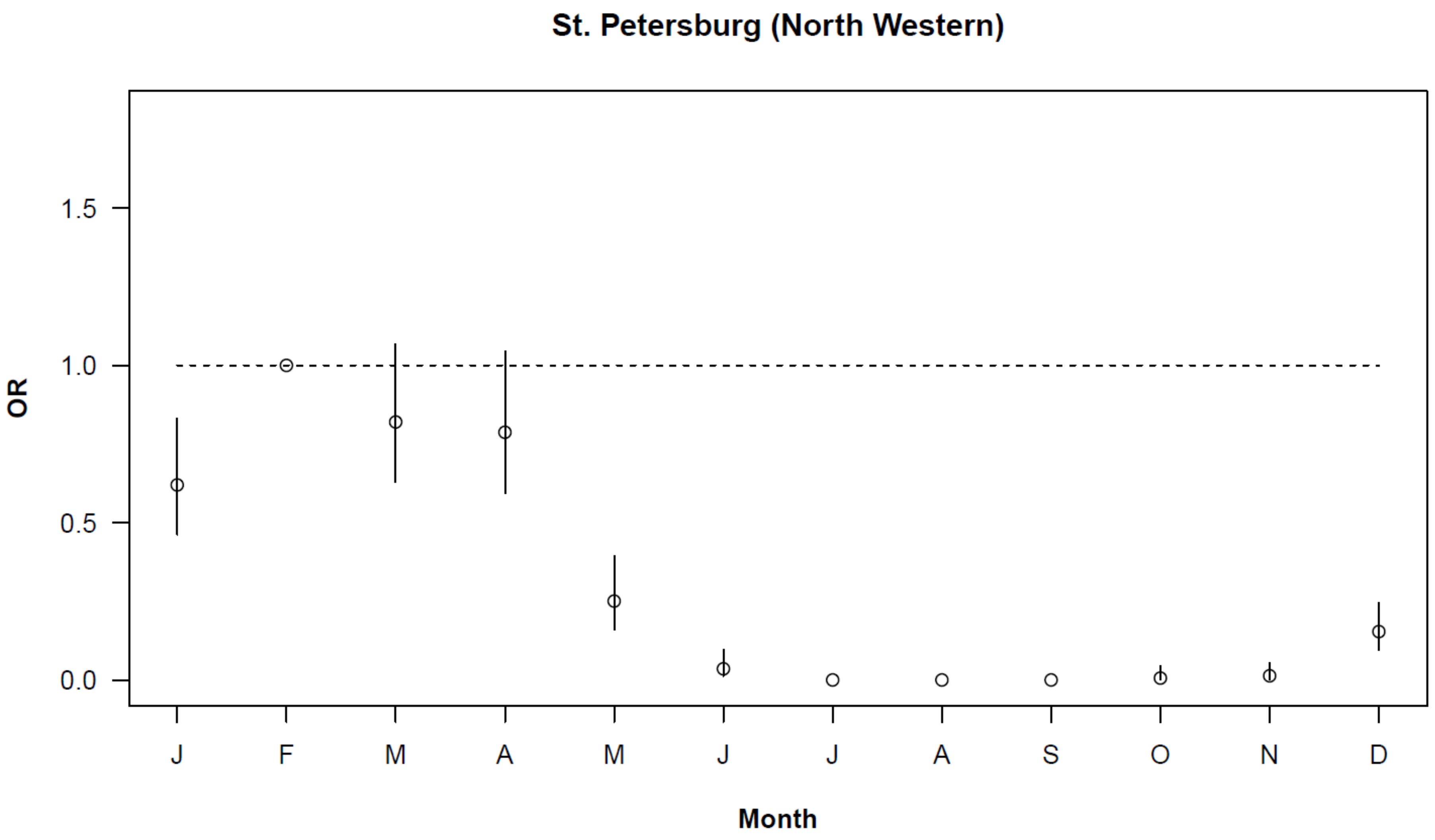

St. Petersburg (North Western)
1.5
1.0
0.5
0.0
OR
J
F
M
A
M
J
J
A
S
O
N
D
Month

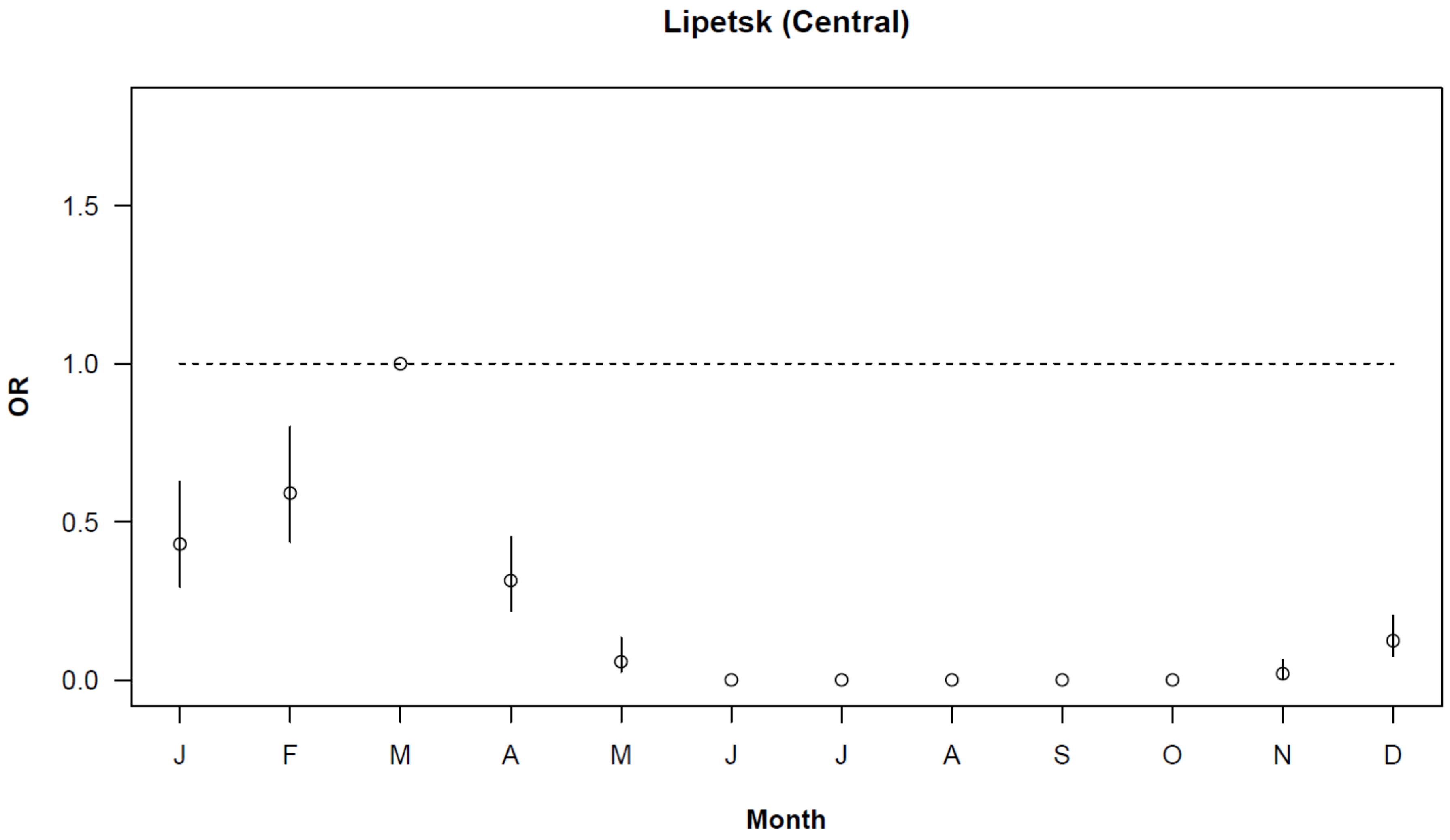
Lipetsk (Central)
OR
1.5
1.0
0.5
0.0
J
F
M
A
M
J
J
A
S
O
N
D
Month

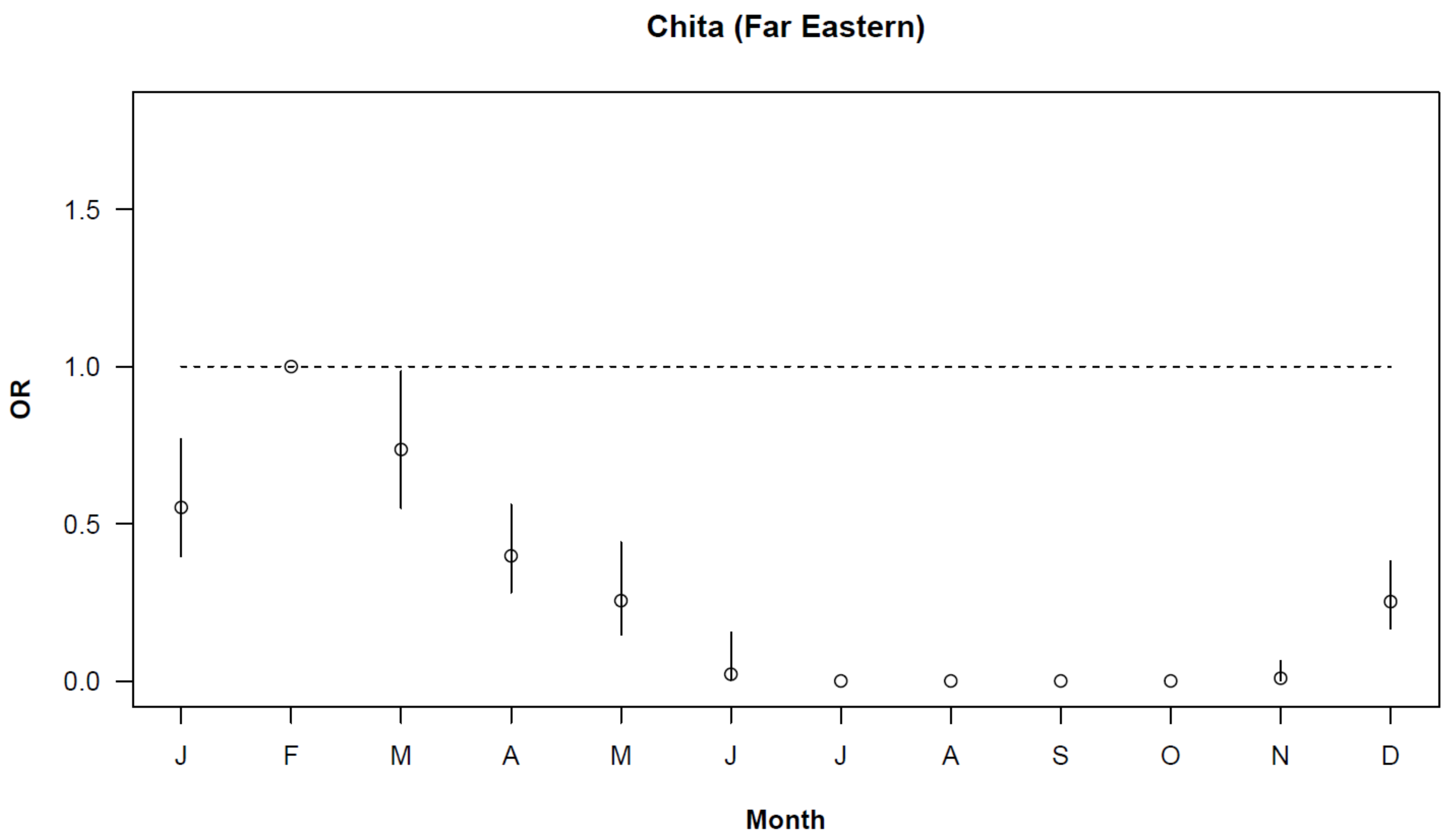
Chita (Far Eastern)
OR
1.5
1.0
0.5
0.0
J
F
M
A
M
J
J
A
S
O
N
D
Month

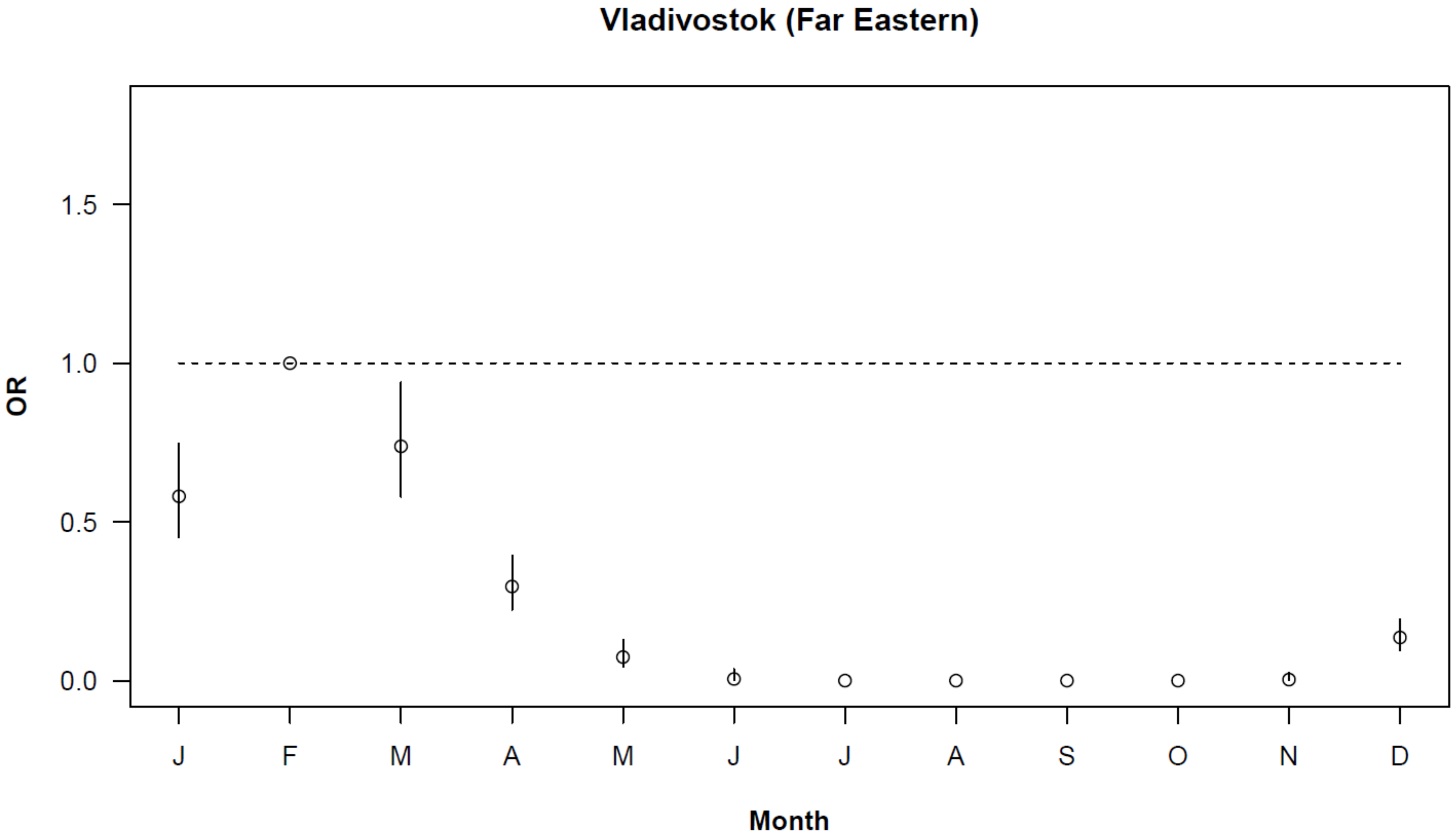

Vladivostok (Far Eastern)
OR
1.5
1.0
0.5
0.0
J
F
M
A
M
J
J
A
S
O
N
D
Month

Figure S8. Seasonality of respiratory syncytial virus (RSV) epidemics in each surveillance site in Russia (January 2014 to April 2019): Kaliningrad (panel a), St. Petersburg (panel b), Lipetsk (panel c), Chita (panel d), and Vladivostok (panel e). Odds ratio (OR) with corresponding 95% confidence intervals for having an RSV-positive test in each month compared to the peak month (reference).

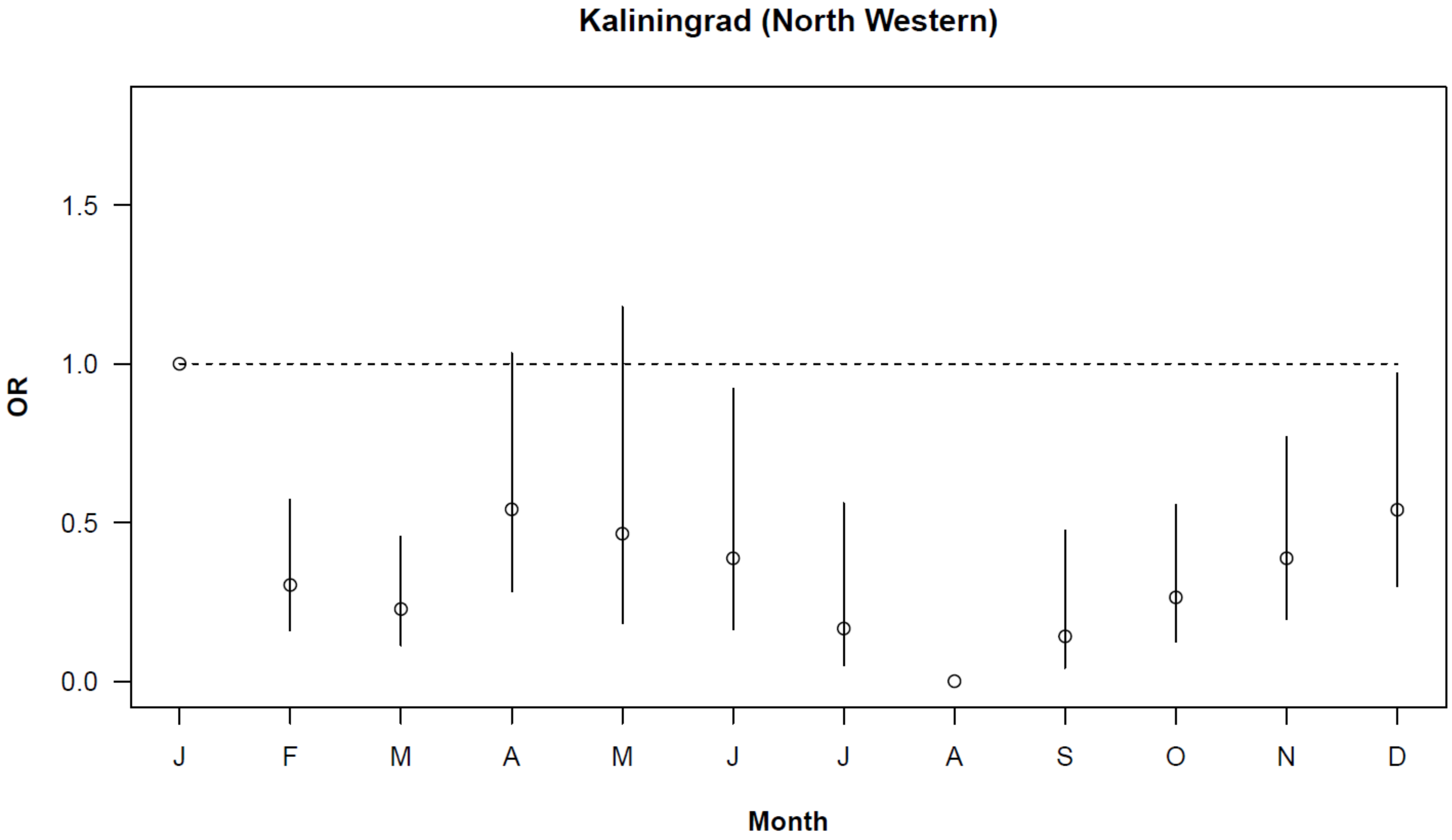

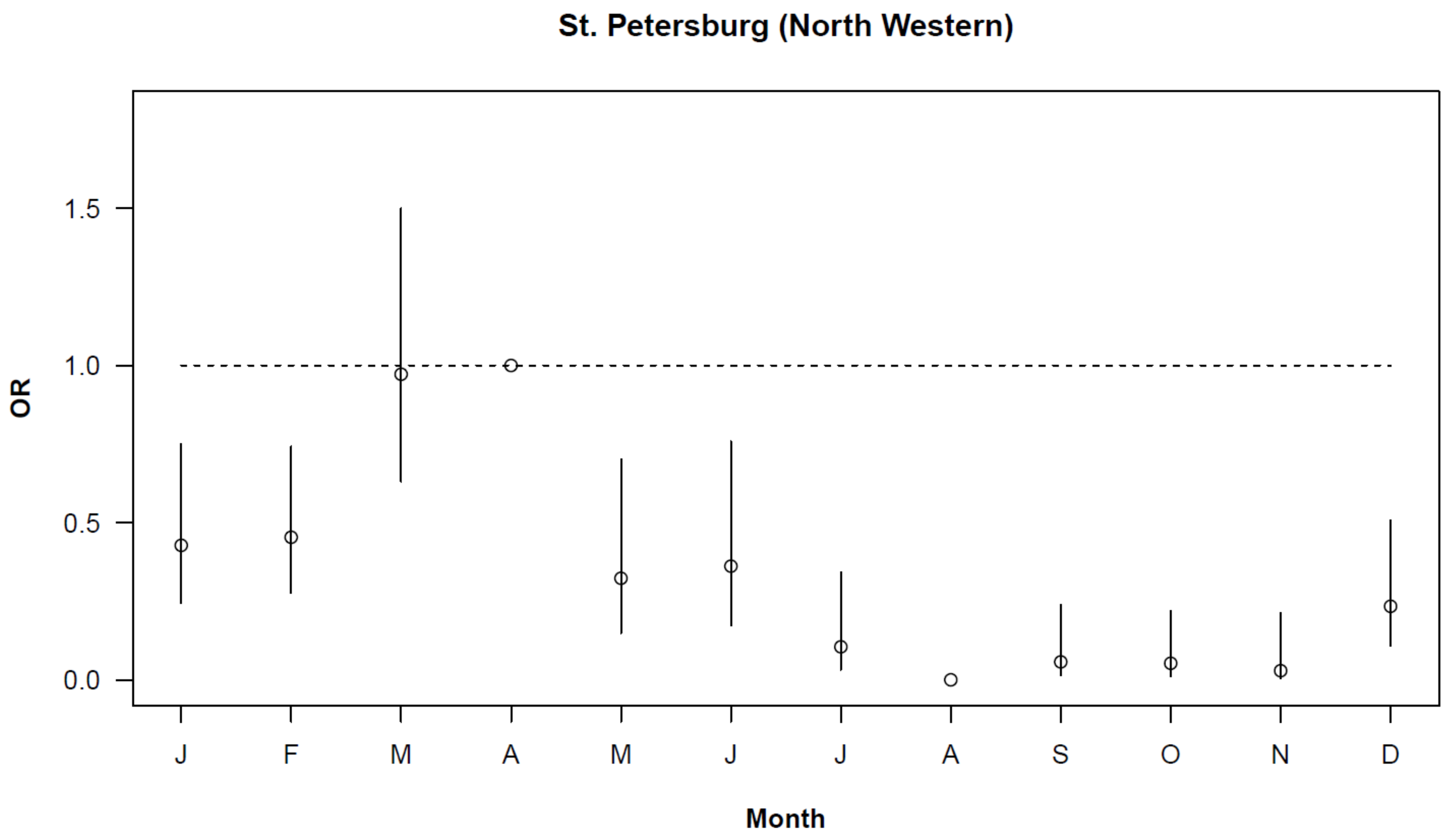
St. Petersburg (North Western)
OR
1.5
1.0
0.5
0.0
J
F
M
A
M
J
J
A
S
O
N
D
Month

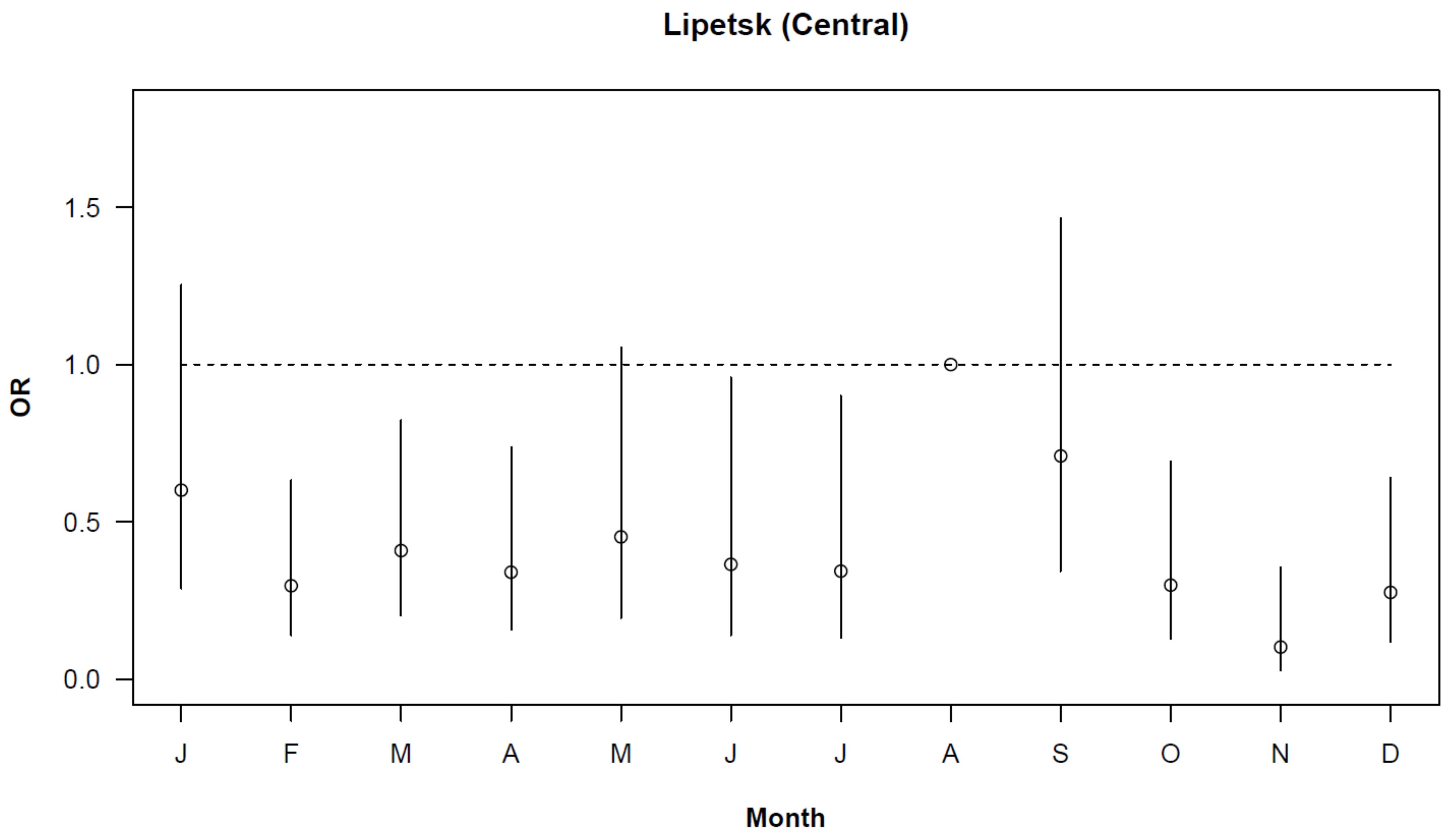
Lipetsk (Central)
OR
1.5
1.0
0.5
0.0
J
F
M
A
M
J
J
A
S
O
N
D
Month

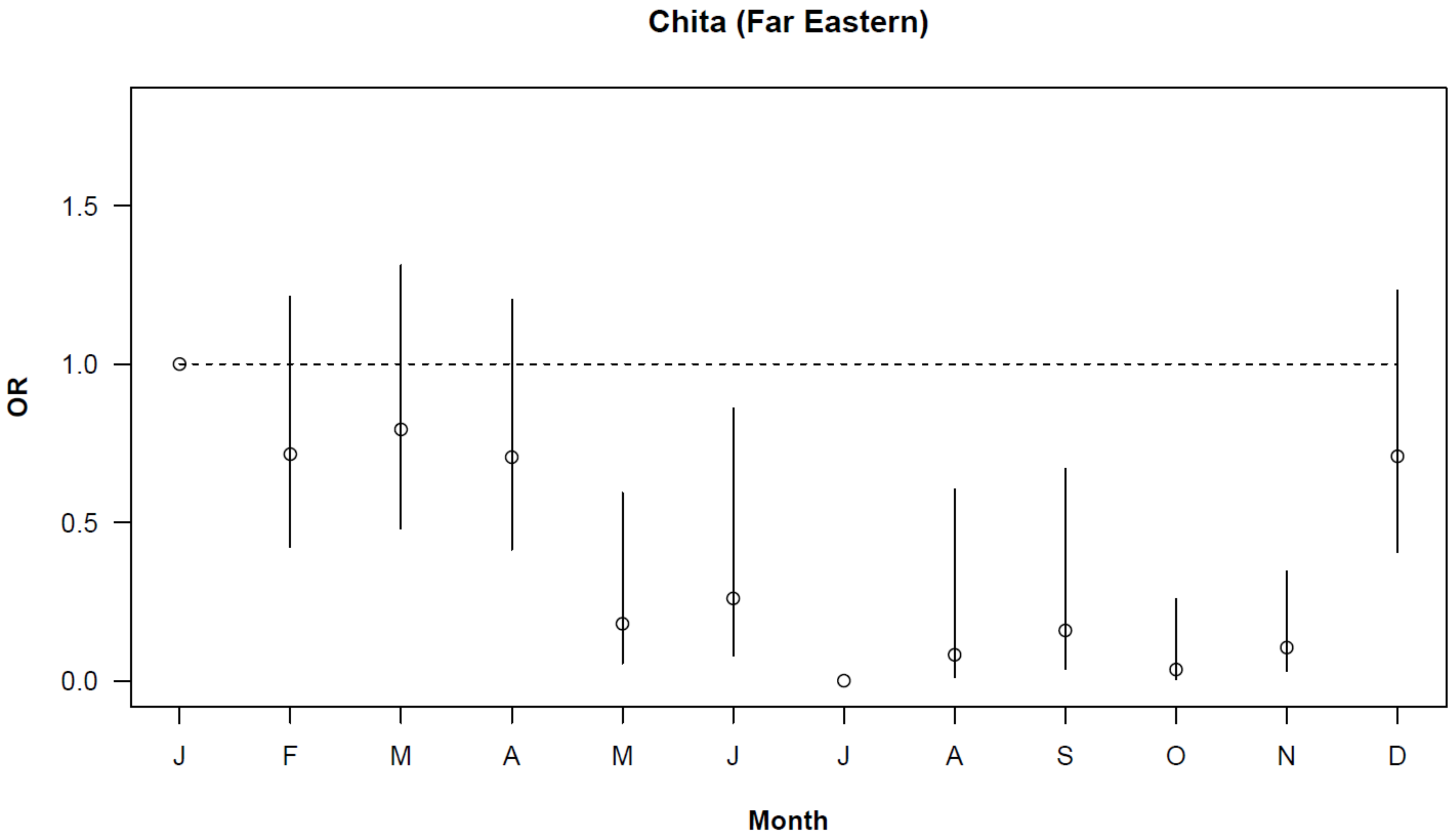
Chita (Far Eastern)
OR
1.5
1.0
0.5
0.0
J
F
M
A
M
J
J
A
S
O
N
D
Month

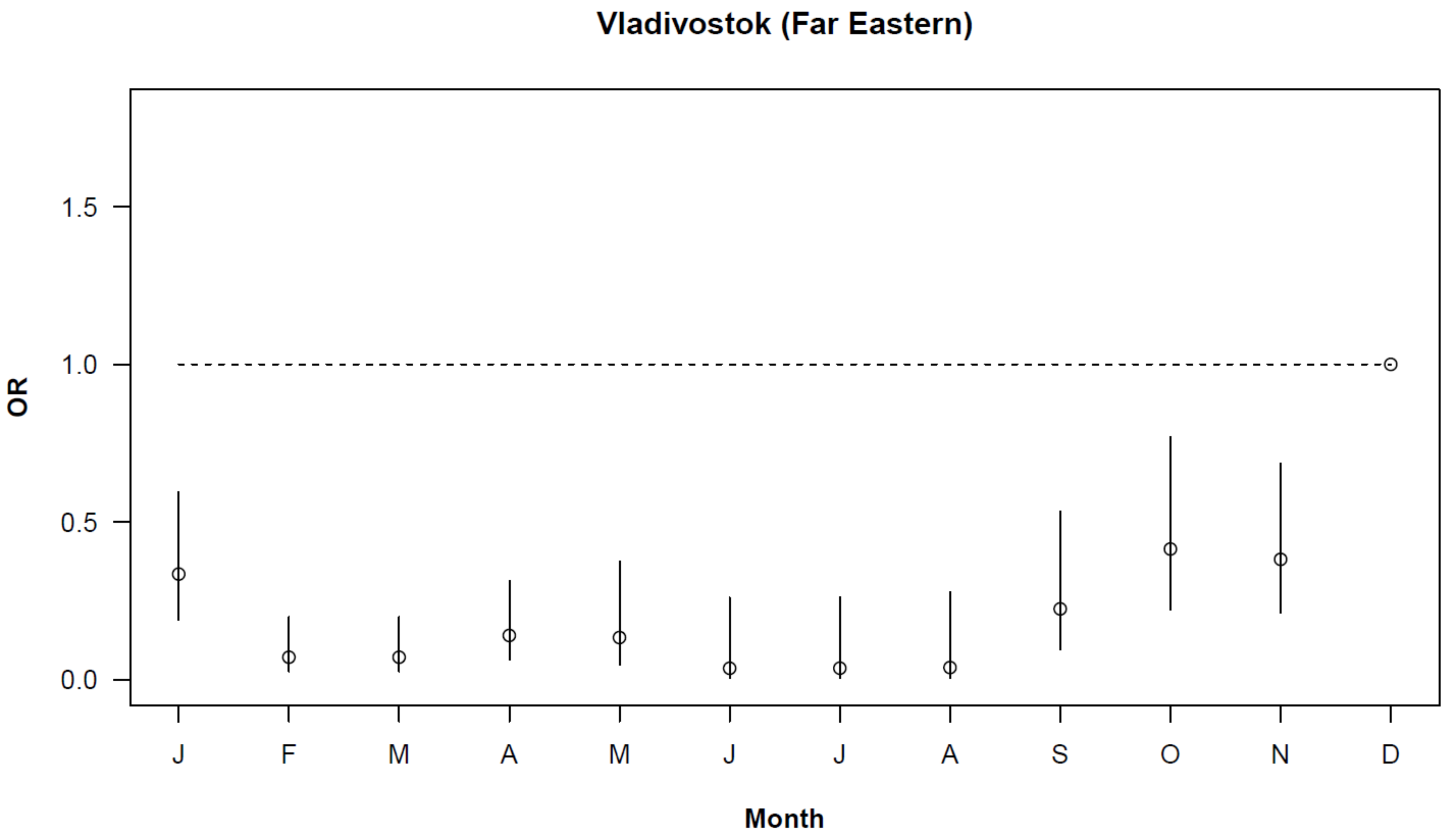

Vladivostok (Far Eastern)
1.5
1.0
0.5
0.0
OR
J
F
M
A
M
J
J
A
S
O
N
D
Month
